# Developing a novel measure of non-rigid, ductile spatial skill

**DOI:** 10.1186/s41235-025-00621-w

**Published:** 2025-03-26

**Authors:** Grace Bennett-Pierre, Thomas F. Shipley, Nora S. Newcombe, Elizabeth A. Gunderson

**Affiliations:** 1 CIRES Center for Education, Engagement, and Evaluation, University of Colorado Boulder, Boulder, CO USA; 2 Department of Psychology and Neuroscience, Temple University, Philadelphia, PA USA; 3 Department of Brain and Psychological Sciences, Indiana University Bloomington, Bloomington, IN USA

**Keywords:** Spatial cognition, Spatial activities, STEM learning, Gender

## Abstract

Non-rigid spatial thinking, or mental transformations where the distance between two points in an object changes (e.g., folding, breaking, bending), is required for many STEM fields but remains critically understudied. We developed and tested a non-rigid, ductile spatial skill measure based on reasoning about knots with 279 US adults (*M* = 30.90, SD 5.47 years; 76% White; 48% women). The resultant 54-item measure had good reliability (*α* = .88). Next, 147 US adults (*M* = 20.65, SD 2.80 years; 48% White; 56% women) completed existing spatial skills measures, the knot reasoning measure, a verbal skill measure, and surveys of current and childhood spatial activities. Knot reasoning performance was significantly, positively correlated with existing measures of spatial skill. Mental rotation and paper folding, but not bending, predicted knot reasoning task performance. We replicated work showing that men performed better than women on mental rotation and unexpectedly found that men also outperformed women on paper folding and knot reasoning, but not bending, tasks. Using structural equation modeling, we found several significant mediation effects. Men who reported less masculine-stereotyped spatial activity engagement had higher performance on the mental rotation and knot reasoning tasks. Women who reported greater engagement in feminine-stereotyped spatial activities had higher paper folding and backwards knot reasoning performance. Spatial skills did not differ among math-intensive STEM, non-math-intensive STEM, and non-STEM majors. The studies introduce a reliable measure of non-rigid, ductile string transformations and provide initial evidence of the role of gender and gendered spatial activities on non-rigid spatial skills.

## Introduction

Spatial thinking, considering the “shapes, locations, paths, relations among entities and relations between entities and frames of reference” (Newcombe & Shipley, 2015, p. 2), underlies both daily tasks (e.g., navigating through a city) and academic pursuits, (e.g., using a number line, understanding geometry, interpreting graphs). Spatial and math skills are linked starting in early childhood (Johnson et al., 2022; Mix & Cheng, 2012; Verdine et al., 2017). Even controlling for concurrent and later math skills, fourth-grade spatial skills predict STEM major choice (Tian et al., 2023). Given the important educational consequences of spatial skills, much research has focused on understanding their nature (e.g., Atit et al., 2013; Harris et al., 2013; Resnick & Shipley, 2013; Vandenberg and Kuse, 1978) and structure (e.g., Mix et al., 2018; Newcombe & Shipley, 2015). However, we lack consensus on the defining distinctions within spatial skills and confirmation that we have developed ways to measure the breadth of critical spatial skills. Reasoning about transformations of non-rigid objects is particularly understudied. Importantly, experimental studies show that spatial skills can be improved in both children and adults through experiences with spatial language and activities (Hawes et al., 2022; Mix et al., 2021), making them a target for intervention. In the present studies, we identify and measure a type of non-rigid spatial thinking that to our knowledge has not been measured before: non-rigid, continuous change in a line or tube. Next, we examined the relationship between existing activities—fiber arts—and performance on new and existing spatial skills measures. Finally, given documented gendered patterns of spatial experience and spatial skill, we investigated whether gendered spatial experiences mediated the relation between gender and performance on rigid and non-rigid spatial skills measures.

### The structure of spatial skills

Despite the importance of spatial skills, both conceptual and methodological factors limit our ability to understand the potentially nuanced pathways from spatial skills to STEM learning. First, there is a lack of consensus in how we should categorize spatial skills. A long tradition of factor analytic approaches has investigated the distinctions within the broader category of spatial skills (see review in Hegarty & Waller, 2005). However, as Hegarty and Waller (2005) point out, not all factor analytic approaches converged on this same structure, and factor analytic approaches are inherently limited by the measures used to assess spatial skills. A more recent theory-driven approach suggests a 2 × 2 framework—dividing spatial skills into intrinsic (within object relations) versus extrinsic (between object relations) and static (stationary) versus dynamic (moving) skills—which draws from linguistics and neuroscience (Chatterjee, 2008; Newcombe & Shipley, 2015). A challenge for both factor analytic and theory-driven approaches is reliance on existing measures of spatial skills that may not capture the full landscape of human spatial thinking.

Against a rich backdrop of spatial skills research, in the present studies we focus on one proposed dimension: the distinction between rigid and non-rigid spatial thinking (Atit et al., 2013; Resnick & Shipley, 2013). The number and type of possible spatial transformations are constrained by the physical materials being transformed: a wooden block allows fewer possible transformations than a piece of string (Santos et al., 2019). Described more precisely, in rigid transformations, the distance between two points within an object does not change (e.g., rotation of a hard object, like the wooden block). In non-rigid transformations, the distance between two points in an object *does* change, either because of a piecewise or brittle transformation (e.g., folding paper, breaking a rock), or a continuous transformation (e.g., bending of a sheet or a string) (Atit et al., 2013). We employ the materials term “ductile,” which refers to materials that can bend without breaking, to refer to the collection of transformations that are applied continuously without local regions of change (e.g., folding) or breaking. Measures of rigid mental rotation that rely on mental rotation of 2-D or 3-D blocks are commonly used to assess spatial skill and are even a part of standardized cognitive assessments like the Block Design Subtest of the Wechsler Preschool and Primary Scale of Intelligence (WPPSI) (Wechsler, 2008). By contrast, non-rigid spatial transformations have been much less studied than their rigid counterparts. The paper folding task (Ekstrom, 1976) is one of the few long-standing measures of non-rigid spatial skill, and, notably, it is a measure of piecewise rigid transformations. Some recent work has focused on another, brittle piecewise transformation—breaking—using images of words that have been broken and pieces of letters moved in differing directions (Resnick & Shipley, 2013). Another recent approach to assessing non-rigid reasoning asked participants to identify shapes that had been transformed through reflection on a three-dimensional mirror (e.g., a cylinder) (Maheshwary et al., 2022). Only one study, to our knowledge, has investigated a non-rigid, *ductile* spatial skill: Atit et al. (2013) developed a measure of mental bending, in which participants had to reason about planar, continuous change (the bending and unbending of a clear plastic sheet). We argue that it is important to, at minimum, check that findings about rigid objects apply to non-rigid objects as well. Further, we will make the case that there could be different relationships between non-rigid spatial thinking and STEM learning, or between gendered spatial experiences and non-rigid spatial thinking than there are for rigid spatial thinking.

In addition to studying non-rigid spatial thinking as a whole, we also argue that there remains another type of non-rigid, ductile spatial skill that has not yet been measured at all: reasoning about *ductile change in a line or tube* (e.g., bending a copper wire or string). We propose that ductile change in a line or tube is unique because it requires thinking about spatial relationships that are either not possible with or are substantively different from the same relationships in rigid or planar materials. For example, when tying a knot with a piece of string, you can think about one or both ends of the string traveling “around” and then “through” other parts of that string—the same object is manipulated continuously without any breaks. In contrast, a single rigid object (or multiple rigid objects) cannot be transformed “through” itself without breaking (a brittle, non-rigid transformation). Even a non-rigid, planar material (e.g., a sheet of paper) cannot travel “through” itself without a brittle, non-rigid transformation (e.g., tearing, cutting) or becoming string- or tubelike (e.g., being rolled into a cylinder). In other words, string allows for continuous change in three dimensions with emergent qualitative spatial properties, and reasoning about such changes has not been studied.

### Why we need to know about non-rigid spatial skills

#### Testing theoretical frameworks

The asymmetry in research on rigid and non-rigid spatial skills poses a problem for basic science questions about the structure of spatial skills. To test the theory that rigid and non-rigid spatial skills are distinct from one another, we need to be able to measure the full breadth of these skills (Maheshwary et al., 2022; Newcombe & Shipley, 2015). Currently, there is only one study that measures non-rigid, *ductile* spatial transformations, and this study included only planar materials (Atit et al., 2013). Given the lack of non-rigid spatial skills measures, it is perhaps not surprising that there has been limited investigation of the rigid versus non-rigid distinction. However, others have tested theoretical distinctions within spatial skills (e.g., extrinsic versus intrinsic) using confirmatory factor analysis (CFA), which provides a quantitative method for adjudicating between different theories (e.g., Mix et al., 2018). Currently, we are not able to adequately test the rigid versus non-rigid distinction because we have too few measures of non-rigid spatial skill. Furthermore, of the non-rigid measures that we do have, most test brittle non-rigid spatial reasoning. Developing measures of ductile, non-rigid spatial skills will allow us to both measure an understudied facet of spatial thinking and lay the groundwork for future researchers to empirically test the rigid versus non-rigid theoretical distinction.

#### Supporting STEM learning about non-rigid phenomena

Beyond basic research questions, a lack of measurement tools for non-rigid spatial skills negatively impacts applied research. Limited existing research on non-rigid skills offers suggestive but incomplete evidence that experts in fields that require thinking about non-rigid phenomena—including geologists, atmospheric scientists, oceanographers, and surgeons—may be better at non-rigid spatial skills than experts in other STEM fields. For example, geologists must reason about the intersecting paths of tectonic plates as they converge and diverge over Earth history, atmospheric scientists and oceanographers study the movement of fluids including currents that pass over and under each other such as the Global Meridional Overturning Circulation (the great ocean conveyor belt), and surgeons must reason about both deformable internal organs and sutures used to repair them (e.g., knots). Geologists and chemists perform similarly on a measure of rigid mental rotation, but geologists—who must frequently reason about rocks bending and breaking—outperform chemists on a measure of breaking, a non-rigid, brittle mental transformation (Resnick & Shipley, 2013). Notably, experts in STEM fields focused on non-rigid phenomena still seem to be good at rigid spatial skills (see McNeal et al., 2019 for disembedding in meteorologists, Brandt & Davies, 2006; Keehner et al., 2004 for rigid mental rotation in surgeons). Together, these studies suggest that gaining a better understanding of non-rigid spatial skills may be important for supporting learning in STEM fields that require thinking about non-rigid phenomena.

#### Creating a clearer picture of gender differences and similarities in spatial skills

Decades of research show a pattern of gender differences in performance on some spatial skills but similarities in performance on others. The mental rotation task (Vandenberg & Kuse, 1978) and some other measures of rigid mental transformation and rotation (e.g., Children’s Mental Transformation Task [CMTT], Levine et al., 1999; Primary Mental Abilities-Space Relations [PMA-SR], Thurstone & Thurstone, 1947) routinely have the strongest and most reliable gender differences in performance, with men and boys generally outperforming women and girls (Lauer et al., 2019; Voyer et al., 1995). Gender differences in mental rotation performance (including on the mental rotation task) emerge by age six and grow with time (Lauer et al., 2019). By contrast, mental folding tasks, including the paper folding task (Ekstrom, 1976) and the Differential Aptitude Test: Spatial Relations Subscale (DAT; Bennett et al., 1947), measures of non-rigid spatial transformation, do not typically show a gender difference in performance (Voyer et al., 1995). Crucially, there is not a clear structural reason within the tasks for why mental rotation and paper folding tasks should elicit different performance in relation to gender (Newcombe, 2020). The existing work using breaking and bending tasks did not explore gender differences or similarities in performance (Atit et al., 2013; Resnick & Shipley, 2013). Despite a general focus on gender *differences* in spatial skills, existing meta-analytic work (Voyer et al., 1995) identifies several spatial skills without reliable gender differences, and it is an open question whether there are gender differences in other, non-rigid, spatial skills. In other words, perhaps men outperform women on a relatively narrow subset of spatial tasks that involve rigid transformations, which may not represent the breadth of spatial skills.

### Environmental exposure shapes spatial skills development and performance

Starting in early childhood, environmental exposure to spatial concepts influences spatial skill. In both naturalistic and experimental settings, preschoolers who hear more spatial language have higher mental rotation and spatial transformation performance (Casasola et al., 2020; Pruden et al., 2011). Beyond spatial talk, parent-reported frequency of four- to seven-year-olds’ play with toys that offer spatial affordances (e.g., blocks, puzzles) was associated with better performance on the WPPSI Block Design task (Jirout & Newcombe, 2015). College students who self-reported greater engagement in spatial activities as a child had higher math scores and higher scores on one measure of spatial skill (water level task, in which participants had to estimate the level of water in various tipped containers), but not on the mental rotation task (Doyle et al., 2012).

Beyond childhood, adult engagement in spatial activities is also related to spatial skill. The Spatial Activities Questionnaire (SAQ), developed in 1983 by Newcombe, Bandura and Taylor, took a list of 231 activities and asked college students to rate how spatial they seemed and whether they seemed “masculine-stereotyped” or “feminine-stereotyped.” A separate sample of college students reported how often they participated in 81 identified tasks and completed a folding measure (the DAT, Bennett et al., 1947). Overall spatial activity engagement was significantly correlated with their DAT score, though this seemed driven by the significant relationship between women’s spatial activities and DAT scores, whereas men’s spatial activities were not significantly correlated with their DAT scores (Newcombe et al., 1983). This study did not find significant relations between gendered subscales and DAT performance (Newcombe et al., 1983). A later study shortened the SAQ to 30 items (10 masculine-stereotyped activities, 10 feminine-stereotyped activities, and 10 neutral activities), and found that for women alone, greater engagement in masculine-stereotyped spatial activities was positively correlated with water level task performance (Signorella et al., 1986). In another study, greater engagement in masculine-stereotyped spatial activities was positively correlated with mental rotation task performance for both men and women (Nazareth et al., 2013). Although men outperformed women on the mental rotation task, this relationship was mediated by participants’ frequency of engagement with specifically masculine-stereotyped spatial activities (Nazareth et al., 2013).

Past and current spatial activities engagement may interact. Adolescents’ self-reported current spatial activities engagement predicted their performance on the paper folding task, above and beyond self-reported childhood spatial activities engagement (Peterson et al., 2020). However, the same study found that even accounting for current engagement, childhood engagement predicted adolescents’ Spatial Habits of Mind, or self-reported tendency to recognize patterns, use spatial descriptions, visualize, use spatial concepts, and use spatial tools. Further, childhood activities predicted adolescent activities. Crucially, this suggests that continued spatial activities engagement—beyond early and middle childhood—may impact spatial skill, though conclusions about a causal relationship are challenging to establish with self-report methods.

### Gendered spatial experiences: a case for fiber arts

Gender influences the spatial input children receive starting early in life. From 34 to 46 months, boys produce more spatial words (e.g., tall, circle, bent) than girls, which is mediated by their parents’ spatial language production at 14 to 26 months (Pruden & Levine, 2017). In turn, children’s spatial language production predicts spatial skills performance later on (Pruden et al., 2011). The types of toys that boys and girls play with are often gendered, and some researchers have argued that feminine-stereotyped toys (e.g., dolls) hold fewer spatial affordances than masculine-stereotyped toys (e.g., blocks, LEGOs) (Voyer et al., 2000). Toy affordances and parent input can also converge. Parents of 2- to 3-year-olds produced more shape names with boys than with girls when playing with the same geometric toys (Verdine et al., 2019). Given the same play materials (Goldiblox, a book and engineering toy designed to be used together), parents of 4- to 6-year-olds spent more time playing with the engineering toy with boys and more time reading the book with girls (Coyle & Liben, 2020). In summary, gendered socialization in the context of spatial language and play starts early in development and altering its trajectory is more complicated than simply increasing girls’ exposure to masculine-stereotyped toys or instructing parents to use spatial terms with their girls.

Work on adult spatial activities engagement identifies feminine-stereotyped spatial activities (e.g., Newcombe et al., 1983; Signorella et al., 1986). Strikingly, in the 30-item version of the SAQ, five of the 10 feminine-stereotyped spatial skills are fiber arts practices (crocheting (with seams), embroidery (no pattern), knitting (multi-color), quilting, and sketching clothes designs) and three of these (embroidery (no pattern), knitting (with seams), and knitting (multi-color)) were positively associated with DAT performance in the original Newcombe et al. (1983) paper. Much less work has focused on identifying feminine-stereotyped spatial activities that occur in childhood and testing whether they are related to spatial skills. Indeed, some researchers have excluded feminine-stereotyped spatial activities in childhood, suggesting that they are too low frequency to be worth measuring (Cherney & Voyer, 2010). We argue that, in addition to increasing girls’ exposure to spatially rich language and toys that have been shown to have spatial affordances, researchers also need to identify what feminine-stereotyped spatial activities exist in childhood. Education researchers, for example, are investigating how fiber arts practices like knitting, crochet, and weaving could be used to support early math and computer science learning (Gresalfi & Chapman, 2017; Peppler et al., 2020), which we have also argued hold affordances for practicing basic spatial skills (Bennett-Pierre & Gunderson, 2023). Importantly for the current studies, the limited existing work on fiber arts provides preliminary evidence that fiber arts activities are associated with at least one non-rigid spatial skill (folding) (Newcombe et al., 1983). We do not know whether feminine-stereotyped spatial activities, including fiber arts, are associated with rigid spatial skills.

In summary, just as research on spatial skills has focused on skills that reliably show a gender *difference* (e.g., mental rotation), research on spatial activities engagement and environmental exposure more broadly has focused on measuring *masculine-stereotyped spatial activities* across the life span and their skill correlates. As a result, we know much more about rigid spatial skills and the activities that support them than we do about non-rigid spatial skills and associated activities. Therefore, an important next step is to both investigate non-rigid spatial skills and investigate feminine-stereotyped spatial activities. From the limited evidence we have, exploring the relation between fiber arts (a feminine-stereotyped activity) and non-rigid spatial skills seems like a productive first step to addressing these gaps. Further, the gendered research imbalance extends to spatial skills interventions. Broadly, there is a focus on getting girls interested in and exposed to the spatial activities that we know promote spatial skills—but those activities are largely masculine-stereotyped (LEGO, blocks, puzzles, etc.) and, notably, rigid in nature (e.g., Casey et al., 2008).

### The current studies

We aimed to answer five research questions. First, we examined the reliability and validity of our new measure (the knot reasoning task) across two samples. Next, we investigated whether performance on existing measures of non-rigid spatial skill (bending task and paper folding task) or rigid spatial skill (mental rotation task) would uniquely predict performance on the new knot reasoning task. We predicted that the bending and paper folding tasks, but not the mental rotation task nor control verbal task, would uniquely predict the knot reasoning task (extending on Atit et al., 2013). Third, we examined whether gender relates to spatial skills and spatial activities engagement. We predicted that men would outperform women on the mental rotation task, but not on the other spatial skills nor the vocabulary task. We also predicted gender-stereotype-consistent endorsement of spatial activities.

Further, we examined whether spatial activities engagement relates to spatial skills and mediates gender differences in spatial skills. We hypothesized that greater current engagement in spatial activities would be positively associated with paper folding performance (replicating Peterson et al., 2020), and that greater engagement in masculine-stereotyped spatial activities would mediate any gendered advantage in spatial skills (e.g., Nazareth et al., 2013). We also tested two exploratory hypotheses: First, that greater current engagement in spatial activities would be positively associated with bending and knot reasoning task performance, and second, greater engagement in feminine-stereotyped spatial activities would be positively associated with all three non-rigid spatial skills (paper folding, bending, and knot reasoning). Finally, we hypothesized, consistent with prior literature (e.g., Tian et al., 2023; Tsigeman et al., 2023), that STEM majors would have higher spatial skills (specifically scores on the mental rotation task and the paper folding task) than non-STEM majors.

Materials, data, and analysis scripts for both studies are available on OSF: https://osf.io/x9uqe/?view_only=84f43a20dcf848738e9f70f2caff17c1. We used R Studio and the lavaan package to complete analyses (R Core Team, 2021; Rosseel, 2012).

## Study 1

### Method

#### Participants

We recruited 300 adult participants from the USA using the online data collection platform Prolific. We determined sample size based on a minimum number of participants needed to conduct item response theory (IRT) analyses. We excluded participants who answered more than half of the attention check questions incorrectly (*n* = 19) or returned the study on Prolific (e.g., withdrew) (*n* = 2), resulting in an analytic sample of 279 participants. Of the analytic sample, 134 participants identified as women, 135 identified as men, and eight identified as non-binary. We recruited a sample of 18- to 40-year-olds (*M* = 30.90, SD 5.47 years) that reflected the 2015 US Census based on race^1^ and sex.^2^ In the analytic sample, 76% of participants were White, 13% were Black or African-American, 5% were Asian or Asian-American, 4% identified as more than one racial group, 1% were Native American, and 1% selected “Other” to describe their race. Participants reported a range of annual income (*M* = $57,650, *SD* = $29,050) and highest level of education (*M* = 14.59, SD 2.01 years).

#### Procedure

Participants completed the study session at home on their computers. To reduce differences in presentation format among devices, we restricted the study to participants using a desktop or laptop computer. Participants saw 73 knot reasoning items of three types (backwards reasoning, different materials, and pulling items), presented in three blocks. Block order, item order within blocks, and answer choices for each item were randomized. We included two attention check questions per block, in which we repeated one of the items with instructions to select one of the responses (e.g., “the fourth photograph below”). We did not impose a time limit on the task, and there were no practice items. On average, the task took 26.43 min (SD 14.66 min; range 3.38–91.37 min) for participants to complete,^3^ and they received $5 for their participation.

#### Materials

##### Knot reasoning task

**Fig. 1:**
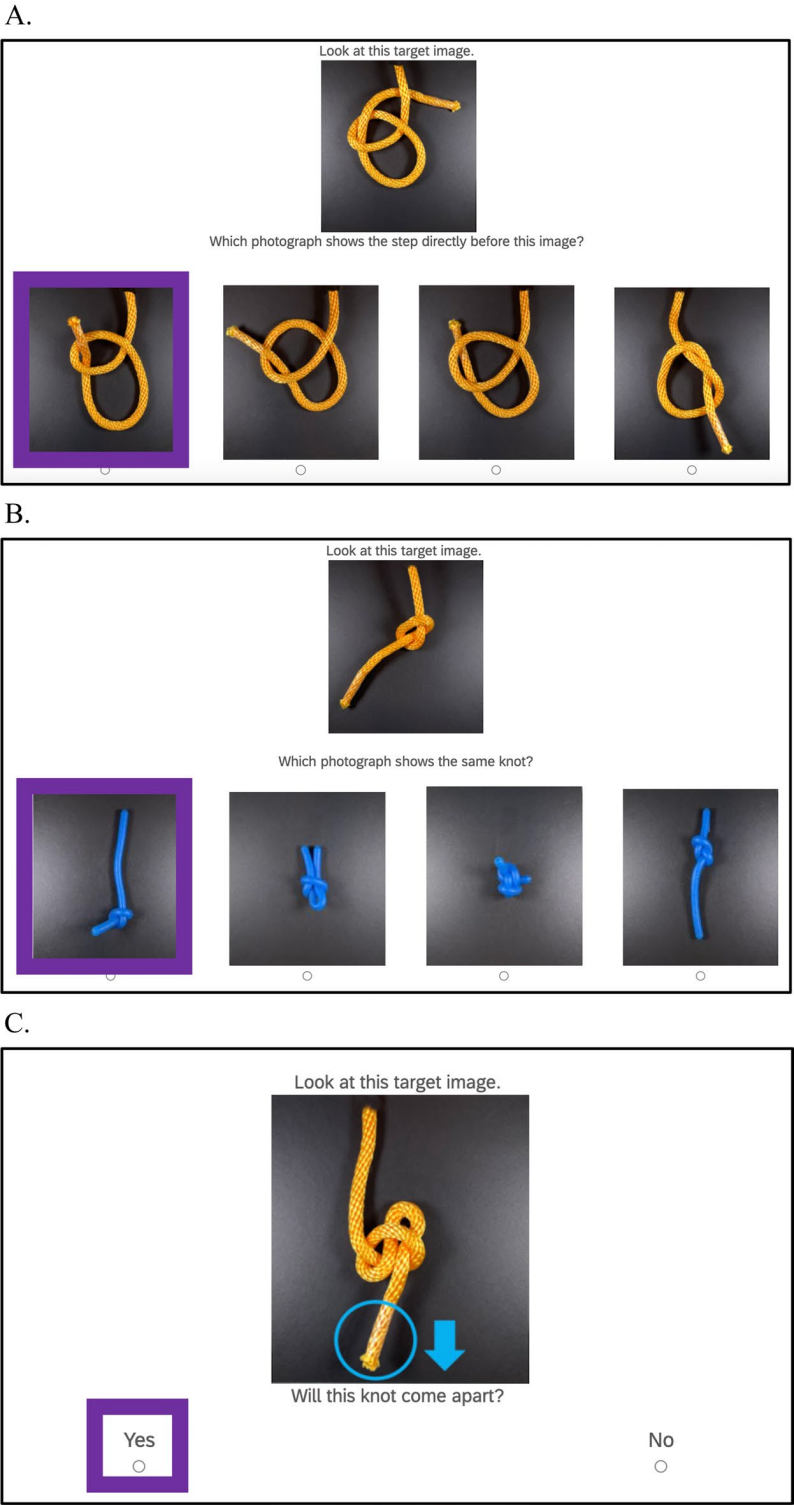
Example items from the knot reasoning task. *Note.* This figure shows the three types of items in the knot reasoning task, **a** backwards reasoning items, **b** different materials items, and **c** pulling items. Correct responses are in purple boxes

***Backwards reasoning items*** Participants completed 28 items in which they looked at a target picture of one step of tying a knot and identified which of four pictures showed the step that came directly before the target (for a sample item, see Fig. 1a; for full task instructions, see Appendix A). We selected knots from a website that gives step-by-step instructions for knot tying (Grogono, 1996), then replicated, and photographed each step. For most knots, a step corresponded to a new crossing of the rope or string. Two of the incorrect responses were the step prior to the target with one crossing switched, making them perceptually similar to the correct response. The final foil was a step from a different knot. Participants received a score of 1 for each correct response and a 0 for each incorrect response. We then calculated a mean accuracy score for each participant.

***Different materials items*** Participants completed 19 items in which they looked at a target picture of a knot in one material (e.g., heavy yellow rope) and identified which of four pictures showed the same knot in a different material (e.g., yarn) (Fig. 1b). The correct response was the same knot and received a score of 1, while the three incorrect responses were different knots and received a score of 0. We calculated a mean accuracy score for each participant.

***Pulling items*** Participants completed 26 items where they determined whether or not a knot would come apart if one of the ends was pulled (Fig. 1c). The knot end and the direction of the pull were indicated with a superimposed circle and arrow. We manually tested whether each knot would come apart to determine the correct answer. Correct responses were scored as a 1 and incorrect responses were scored as a 0. We calculated a mean accuracy score for each participant.

### Results

Descriptively, collapsing across all three item types, participants answered most questions correctly (*M* = 0.71, SD 0.12). Performance varied across subscales, with backwards reasoning items having the lowest performance (*M* = 0.59, SD 0.18), different materials items falling in the middle (*M* = 0.74, SD 0.12), and pulling items having the highest performance (*M* = 0.81, SD 0.13). We report task performance by gender in Table 1. We compared men’s and women’s performance with t-tests, and found that performance did not significantly differ, except for the different materials subscale, on which women performed significantly better than men (Table 1; *t*(266.98) = 3.08, *p* = 0.002, *d* = − 0.38). We did not run statistical comparisons with the non-binary participants because the size of this group was much smaller than the other two gender groups.

**Table 1:** Study 1 knot reasoning task performance by gender

Variable	Men (*n* = 135)	Women (*n* = 134)	Non-binary (*n* = 8)
	*M* (SD)	*M* (SD)	*M* (SD)
Overall	0.69 (0.12)	0.71 (0.12)	0.81 (0.10)
Backwards Reasoning	0.59 (0.18)	0.59 (0.19)	0.71 (0.20)
Different Materials	0.72 (0.12)	0.76 (0.12)	0.82 (0.11)
Pulling	0.79 (0.14)	0.82 (0.12)	0.91 (0.05)

We conducted an item response theory (IRT) analysis to evaluate the knot reasoning task’s reliability. We first ran a one parameter model (1PL), fixing the second, discrimination parameter (a) to 1, and the third, guessing parameter (c) to 0.25 (backwards reasoning and different materials) or 0.5 (pulling) depending on the type of question. Next, we ran a two-parameter model (2PL), allowing both the difficulty parameter (b) and the discrimination parameter (a) to vary (Appendix B, Table 7). The 2PL model fit the data significantly better than the 1PL model (*X*^2^ = 246.41, *p* < 0.001), indicating that allowing the discrimination parameter to vary explained the data better even accounting for the differences in degrees of freedom. Next, we removed the 19 items with a discrimination parameter (a) less than 1 in the 2PL model and reran the 2PL model using only the remaining 54 items (Appendix B, Table 8).^4^ Overall scale reliability for the 54 items was *α* = 0.88 and reliability was greater than 0.80 for participants who were within two standard deviations below and above the mean. We did not have enough participants to run a 3PL model (DeMars, 2010).

**Table 2:** Descriptive statistics for cognitive and activities scores

Variables	All participants (*n* = 147)	Men (*n* = 56)	Women (*n* = 82)	Non-binary people (*n* = 9)
	*M* (SD)	*M* (SD)	*M* (SD)	*M* (SD)
MRT	19.31 (11.05)	24.14 (10.64)	15.93 (10.38)	20.11 (8.82)
PFT	10.86 (3.97)	11.66 (3.97)	10.21 (3.97)	11.88 (3.10)
BT	.73 (.12)	0.75 (0.11)	.73 (.12)	.71 (.17)
KRT	.84 (.10)	0.86 (0.10)	.82 (.10)	.90 (.06)
KRT—BR	.76 (.18)	0.80 (0.17)	.73 (.18)	.79 (.12)
KRT—DM	.92 (.09)	0.93 (0.07)	.91 (.10)	.94 (.06)
KRT—P	.87 (.11)	0.88 (0.11)	.85 (.11)	.96 (.05)
WS	.68 (.15)	0.71 (0.15)	.65 (.14)	.74 (.15)
SAQ	1.36 (.24)	1.31 (0.22)	1.40 (.23)	1.34 (.30)
SAQ masculine	1.17 (.22)	1.30 (0.28)	1.10 (.14)	1.08 (.12)
SAQ feminine	1.29 (.25)	1.12 (0.15)	1.41 (.23)	1.35(.36)
C-SAQ	3.25 (1.68)	2.93 (1.57)	3.43(1.73)	3.53 (1.84)
C-SAQ masculine	2.03 (2.74)	3.77 (3.57)	.97 (1.30)	1.28 (1.20)
C-SAQ feminine	2.36 (2.09)	.90 (1.38)	3.30 (1.91)	2.96 (2.27)

*MRT* Mental rotation task (possible range: − 40 to 40), *PFT* paper folding task (possible range: − 4 to 20), *BT* bending task (possible range: 0–1), *KRT* knot reasoning task (possible range: 0–1) and subscales, *BR* backwards reasoning, *DM* different materials, *P* Pulling, *WS* Wordsum Plus (possible range: 0–1), *SAQ* Spatial Activities Questionnaire (possible range: 1–6), *SAQ masculine* Spatial Activities Questionnaire Masculine-Stereotyped Items (possible range: 1–6), *SAQ feminine* Spatial Activities Questionnaire Masculine-Stereotyped Items (possible range: 1–6), *C-SAQ* Childhood Spatial Activities Questionnaire (possible range: 0–36), *C-SAQ masculine* Childhood Spatial Activities Questionnaire Masculine-Stereotyped Items (possible range: 0–36), *C-SAQ feminine* Childhood Spatial Activities Questionnaire Feminine-Stereotyped Items (possible range: 0–36)

**Fig. 2:**
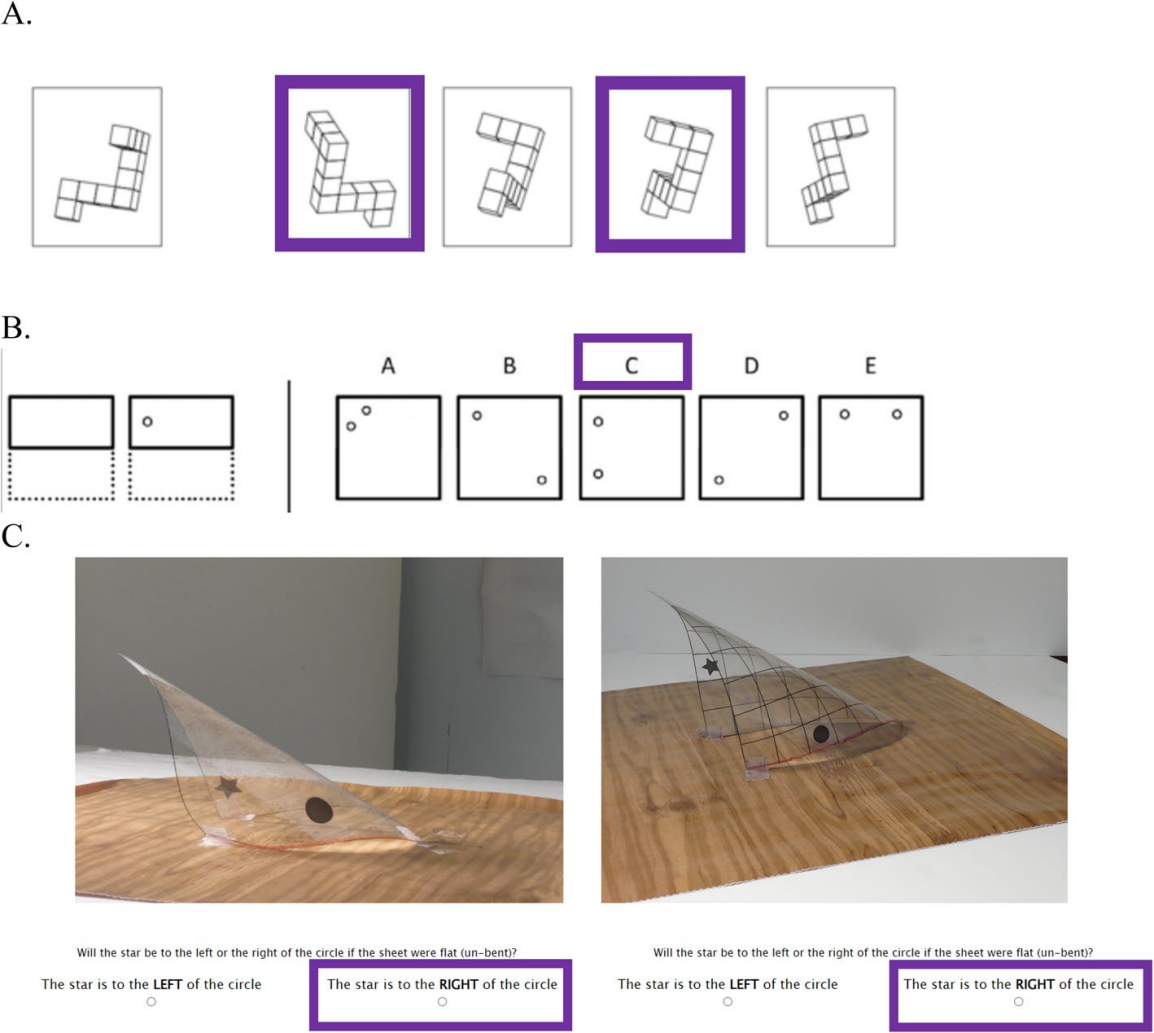
Example items from existing spatial skills tasks. *Note.* This figure shows the three existing measures of spatial reasoning, **a** the mental rotation task (Peters et al., 1995; Vandenberg & Kuse, 1978), **b** the paper folding task (Ekstrom, 1976), and **c** the bending task (Atit et al., 2013). Images reproduced from Fehringer (2020). Note that the exact images for the mental rotation and paper folding tasks varied slightly from those shown here. Correct responses are indicated with purple boxes

## Study 2

In Study 2, we first aimed to establish the reliability of the knot reasoning measure in a second, in-person sample. Next, we tested the relation between the knot reasoning measure and established rigid and non-rigid spatial skills measures. Third, we examined the relations among childhood and current gendered spatial activities engagement and performance on all four spatial skills measures. Finally, we asked whether spatial skills performance varied depending on participants’ college major choice. Study 2 expands our understanding of the structure of spatial skills and the relative contributions of different spatial activities to spatial skills.

### Methods

#### Participants

For Study 2, we recruited 148 adult participants, for an analytic sample of 147.^5^ This sample size was based on a power analysis to achieve power = 0.80 with alpha = 0.05 for a multiple linear regression, with a partial R-squared of 0.07. This effect size estimate was based on a prior study of non-rigid spatial skills (Atit et al., 2013). Further, this sample size would allow us to distinguish between two CFA models with a RMSEA model fit of 0.04 and 0.07 (MacCallum et al., 2006), and aligns with a 10:1 participants-to-free-parameters rule for SEM models (Bentler & Chou, 1987). Participants were recruited at a public mid-Atlantic university, among undergraduates taking Psychology courses, through flyers posted on campus, and through emails sent to student organizations and undergraduate classes focused on STEM or fiber arts. We again limited participant age to 18–40 years and aimed for no more than 60% women in our sample.

In the analytic sample, 82 participants identified as women, 56 identified as men, and nine identified as non-binary or another gender. Forty-eight percent of participants were White, 14% were Black or African-American, 25% were Asian or Asian-American, 7% identified as more than one racial group, and 5% selected “Other” to describe their race. Eleven percent of participants were Hispanic. Participants were, on average, 20.65 years old (SD 2.80). Age did not differ significantly by gender when non-binary participants were included (*F*(2, 144) = 2.66, *p* = 0.073, *η*^2^ = 0.04). However, men were older than women (Men: *M* = 21.3, SD 3.40; Women: *M* = 20.2, SD 2.24; *t*(87.11) = − 2.14, *p* = 0.035, *d* = 0.40).^6^ All of the participants in Study 2 had completed high school, and 89% were in the process of completing an undergraduate degree. Of the 131 participants currently completing an undergraduate degree, there was a fairly even distribution among years (first year: 25%, second year: 33%, third year: 25%, fourth year: 15%, beyond fourth year: 3%). Year in college did not differ by gender when non-binary participants were included (*F*(2,125) = 0.334, *p* = 0.716, *η*^2^ = 0.005), nor when men and women were compared (*t*(103.60) = − 0.80, *p* = 0.428, *d* = 0.15). Most of the remaining 16 participants had completed a Bachelor’s degree (*n* = 6) or part or all of a graduate degree (*n* = 8). One participant completed high school and one completed at least one year of college. We categorized participants’ undergraduate major using a coding scheme developed by Tian et al., (2023) (see Appendix E) More than half of the participants were STEM majors (55%); of those, 71% were non-math-intensive STEM and 29% were math-intensive STEM. An additional three participants were undecided. Participants reported a range of annual income (*M* = $66,440, SD $35,600).

#### Procedure

Participants completed a one-hour session using Qulatrics on a desktop computer in a quiet room with an experimenter. Participants first completed the five cognitive skills tasks—the mental rotation task, paper folding task, bending task, knot reasoning task, and the Wordsum Plus vocabulary test—in a randomized order. Each task except for knot reasoning had a time limit implemented automatically in Qualtrics. All participants completed the Spatial Activities Questionnaire and a demographics questionnaire last. To alleviate participant fatigue, participants had a 5-min break after the first three tasks. Participants were debriefed about the purpose of the study and received course credit or a $30 gift card.

#### Materials

***Knot reasoning task*** Participants responded to the 54 items (20 backwards reasoning items, 12 different materials items, and 22 pulling items) from Study 1 (Fig. 1). We created one mean score. Reliability was acceptable, though lower than in Study 1 (*α* = 0.80).

***Vandenberg and Kuse mental rotation task*** The Vandenberg and Kuse (1978) mental rotation task has three untimed practice items with feedback and 20 timed test items (Fig. 2a). Participants had three minutes for the first 10 items, took a 2-min break, and had three minutes for the final 10 items. Participants specified which two rotated images (out of four) matched the target image and received one point for each correct image selected, and lost a point for each incorrect image selected. For example, if a participant selected both correct images, they received a score of 2 for the item; if they selected one correct and one incorrect, they received a score of 0. If participants gave more than 2 responses, they were given a 0 for that item. Few items were invalidated in this way (0.3%). The maximum overall score was 40, and the minimum score was − 40. Reliability was good (*α* = 0.85) and similar to that found in prior studies (e.g., Atit et al., 2013, *α* = 0.80).

***Paper folding task*** For the paper folding task, participants had three minutes for the first 10 items, had three minutes for the final 10 items, and took a two-minute break in between (Fig. 2b; Ekstrom, 1976). Each item shows a line drawing representing a piece of folded paper with holes poked through it. Participants specified which one (out of five) unfolded pieces of paper matched the folded target. Correct responses earned 1 point and 0.20 points were subtracted for each incorrect response, making the maximum score 20 and minimum score − 4 (Peterson et al., 2020). Reliability was good (*α* = 0.77) and similar to prior studies (e.g., Peterson et al., 2020, *α* = 0.79; Atit et al., 2013, *α* = 0.79).

***Bending task*** The bending task was adapted from Atit et al. (2013). Participants saw pictures of bent, clear plastic sheets that had a star and a circle printed on them in different locations (Fig. 2c). Consistent with the original study, participants had six seconds to indicate whether the star would be to the right or left of the circle on each sheet if the sheet were unfolded. The original task included 85 items separated into two trial types (gridline backgrounds and textured backgrounds) and three levels of difficulty (easy, medium, and hard). To reduce the task’s overall length, we selected a shortened set of 48 “hard” items which showed the most individual variability in the original study. We included 24 of each gridline and texture items, and an equal number of items where “left” and “right” were the correct answer. Participants received one point for each correct answer, and we created a mean score. Reliability was good (*α* = 0.78), though descriptively not as high as the reliability found by Atit et al. (2013) for the larger set with easier items (*α* = 0.98 for gridline items, *α* = 0.95 for texture items).

***Wordsum Plus*** The Wordsum Plus is a 14-item vocabulary questionnaire (Malhotra et al., 2007). Participants saw a target word and had to select the one word of five options with a meaning closest to the target. Participants could also select “don’t know,” which we counted as incorrect. Participants received one point for each correct answer, and we created a mean score, with a minimum value of 0 and a maximum value of 1. Unlike prior studies, we found relatively low reliability for this scale (*α* = 0.58; vs. *α* = 0.79; Cor et al., 2012).

***Short Version of the Spatial Activities Questionnaire*** Participants responded to the 30-item version of the Spatial Activities Questionnaire (Signorella et al., 1986). In line with Signorella et al. (1986), ten items were “masculine-stereotyped” activities, ten items were “feminine-stereotyped” activities, and ten items were “neutral” activities. Participants responded on a six-point Likert scale how often they do each activity, where a 1 represents “never,” and 6 represents “more than once a week.” We added 12 fiber arts activities that expanded on the ones already included in the scale. For example, the original questionnaire asked about “crochet (with seams),” and we added “crochet (without seams)” to capture the full breadth of exposure to fiber arts activities. We also asked about participants’ spatial activities experience in childhood (defined as < 18 years of age) using questions adapted from the SAQ. Participants reported for how long they participated in each of the SAQ activities when they were a child on a scale from never to six or more years. If they participated for 1 or more years in an activity in childhood, they were prompted to report how often they did each activity, where a 1 represents “never,” and 6 represents “more than once a week.” Finally, we asked participants directly about their experience with knot tying through a variety of activities (e.g., Boy or Girl Scouts), on a scale of 1 “None” to 4 “Expert,” and also provided a free response box for participants to report any other sources of knot-tying experience that they had. A full list of activities is reported in Appendix C.

We made three current spatial activities scores for each participant. First, we made an overall spatial activities score by averaging responses to all 42 activities (minimum: 1, maximum: 6).^7^ Reliability of the overall SAQ was acceptable (*α* = 0.70) though lower than prior work using adolescents (Peterson et al., 2020, *α* = 0.82). Next, we made an expanded feminine-stereotyped activities score and a masculine-stereotyped activities score by taking the mean of the 10 feminine-stereotyped activities plus the 12 additional fiber arts tasks (minimum: 1, maximum: 6, *α* = 0.65) and the 10 masculine-stereotyped activities (minimum: 1, maximum: 6, *α* = 0.51).^8^ The reliability of each of these subscales was lower than prior studies (Signorella et al., 1986 found *α* = 0.77 for the feminine-stereotyped subscale and *α* = 0.79 for the masculine-stereotyped subscale).

We also created three childhood spatial activities scores that reflected both duration and intensity information for each of the childhood activities. First, we multiplied the intensity response for each activity (1 = never participated to 6 = participated more than once a week) by the duration response (0 = never participated to 6 = participated for 6 or more years) to yield a new score between 0 and 36.^9^ Then, we took the mean of the new item-level scores to create a childhood spatial activities score for each child (minimum 0, maximum 36). We also created an expanded childhood feminine-stereotyped activity score and a childhood masculine-stereotyped activity score by following the same procedure for these subsets of items. Reliability for the overall childhood SAQ was similar to the current SAQ (*α* = 0.68), as was the reliability for the feminine-stereotyped subscale (*α* = 0.69) and the masculine-stereotyped subscale (*α* = 0.66).

### Results

#### Missing data and data transformations

We anticipated that there could be missing data, because a participant chose to skip a question, ran out of time on timed measures, or chose to end the session early. For the cognitive tasks, a non-response or an “I don’t know” response (Wordsum task only) was scored as incorrect. For cognitive tasks where an incorrect response resulted in a subtraction of points (mental rotation task, paper folding task), a non-response counted as a 0. We planned to exclude participants who had 50 percent or more of responses on the SAQ measure missing but no participants met this condition. We examined the data for normality and skewness and made transformations if there were serious violations of normality. Responses to the knot reasoning task were not normally distributed, so we performed an arcsine transformation on this score. We also standardized all measures before conducting inferential analyses. We checked for outliers (± 3 standard deviations away from the mean, Fein et al., 2022) and excluded participants’ data for a given task if they were an outlier. Eight participants were outliers on at least one task.^10^

#### Preliminary analyses

We report descriptive statistics on all measures overall and by gender (Table 2). Next, we looked at the correlations among the cognitive measures and spatial activities scores (Table 3).

**Table 3:** Correlations among the cognitive and activities scores

	1	2	3	4	5	6	7	8	9	10	11	12	13
1. MRT													
2. PFT	.53***												
3. BT	.31***	.47***											
4. KRT	.54***	.59***	.33***										
5. KRT—BR	.46***	.52***	.32***	.87***									
6. KRT—DM	.36***	.42***	.39***	.62***	.39***								
7. KRT—P	.38***	.39***	.13	.73***	.34***	.39***							
8. WS	.28**	.28**	.04	.29***	.33***	.22**	.07						
9. SAQ	− .10	.00	− .02	− .12	− .11	.04	− .14	.01					
10. SAQ masculine	− .01	− .05	.00	− .07	− .02	.00	− .12	.13	.36***				
11. SAQ feminine	− .18*	.05	.05	.01	.02	.13	− .07	− .07	.69***	− .13			
12. C-SAQ	.05	.11	.16	.05	.02	.13	.04	.05	.51***	.19*	.37***		
13. C-SAQ masculine	.15	.08	.11	.01	− .04	.16	.00	.12	.20*	.70***	− .25**	.35***	
14. C-SAQ feminine	− .19*	− .01	.05	.00	− .05	.06	.03	− .14	.43***	− .22**	.66***	.69***	− .24**

*MRT* Mental rotation task, *PFT* paper folding task, *BT* bending task, *KRT* knot reasoning task (possible range: 0–1) and subscales, *BR* backwards reasoning, *DM* different materials, *P* Pulling, *WS* Wordsum Plus, *SAQ* Spatial Activities Questionnaire, *SAQ masculine* Spatial Activities Questionnaire Masculine-Stereotyped Items, *SAQ feminine* Spatial Activities Questionnaire Masculine-Stereotyped Items, *C-SAQ* Childhood Spatial Activities Questionnaire, *C-SAQ masculine* Childhood Spatial Activities Questionnaire Masculine-Stereotyped Items, *C-SAQ feminine* Childhood Spatial Activities Questionnaire Feminine-Stereotyped Items

***p < .001, **p < .01, *p < .05

##### Reliability and validity of knot reasoning measure

First, we examined the internal reliability and item discrimination of the knot reasoning measure in a second sample. We ran 1PL and 2PL models with the guessing parameter set to 0.25 for the backwards reasoning and different materials items and 0.50 for the pulling items (Appendix B, Table 9). The 1PL and 2PL models did not significantly differ in their fit (*X*^2^(52) = 61.58, *p* = 0.171), indicating that, unlike Study 1, the 2PL model did not explain the data better. Overall scale reliability was *α* = 0.80, which was lower than Study 1 (*α* = 0.88). Reliability was around 0.80 for participants who were two standard deviations below and one standard deviation above the mean.

Next, we assessed the convergent and divergent validity of the knot reasoning task by examining the correlations between the knot reasoning task and previously used cognitive measures (mental rotation task, paper folding task, bending task, Wordsum Plus vocabulary task) (Table 3). Similar to prior work, the mental rotation task, paper folding task, and bending task were significantly positively correlated with one another (e.g., Atit et al., 2013). The new knot reasoning task was also significantly positively correlated with the other spatial tasks (0.33 < *r* < 0.59, *p*s < 0.001). The verbal control measure (Wordsum Plus vocabulary task) was significantly, positively correlated with three of the spatial skills measures (mental rotation task, paper folding task, knot reasoning task, 0.28 < *r* < 0.29, *p*s < 0.002).

##### Relation between existing spatial skills measures and knot reasoning task

We hypothesized that participants’ bending and paper folding task performance would uniquely predict their performance on the knot reasoning task because all three measure non-rigid spatial skills. However, performance on the mental rotation task and paper folding task, but not the bending task, significantly predicted performance on the knot reasoning task in a simultaneous regression (Table 4, Model 1: mental rotation: *β* = 0.26 SE 0.08, *p* = 0.001; paper folding: *β* = 0.39, SE 0.08, *p* < 0.001). Further, the same pattern held when we included age and gender as covariates (Table 4, Models 2 and 3—for model 3, only men and women were included in the model). We then checked whether our data replicated Atit et al.’s (2013) finding that bending task performance was predicted by paper folding, but not mental rotation performance, which it did (mental rotation: *β* = 0.08 SE 0.09, *p* = 0.377, paper folding: *β* = 0.42 SE 0.08, *p* < 0.001).

**Table 4:** Simultaneous regression with knot reasoning task (arcsine-transformed) predicted by other cognitive tasks

	Cognitive measures only	Cognitive measures and demographics	Cognitive measures and demographics—only men and women
	Model 1 (*n* = 144)	Model 2 (*n* = 144)	Model 3 (*n* = 136)
Variable	*β* (SE)	*β* (SE)	*β* (SE)
MRT	.26 (.08)***	.26(.08)**	.30 (.08)***
PFT	.38 (.08)***	.38 (.08)***	.40 (.08)***
BT	.05 (.08)	.06 (.08)	.05 (.08)
WS	.11 (.07)	.11 (.07)	.10 (.07)
Age		.03 (.02)	.04 (.02)
Gender			− .10 (.15)
R-squared	40.48	41.14	44.08
F-stat (df)	23.64*** (4, 139)	19.29*** (5, 138)	16.95*** (6, 129)

*MRT* Mental rotation task—standardized, *PFT* paper folding task—standardized, *BT* bending task—standardized, *KRT* knot reasoning task—arcsine-transformed and standardized, *WS* Wordsum Plus—standardized

**p < .01, ***p < .001

Next we conducted simultaneous regressions with each item type from the knot reasoning task modeled separately, including age and gender as covariates (Table 5). The backwards reasoning and pulling items showed the same pattern as the overall knot reasoning task (Table 5, Models 1, 2, 5, 6). However, the different materials items exhibited a pattern consistent with our prediction, where paper folding and bending, but not mental rotation, predicted knot reasoning performance (Table 5, Models 3 and 4).

**Table 5:** Simultaneous regression with three item types from knot reasoning task (arcsine-transformed) predicted by other cognitive tasks

	Backwards reasoning items	Backwards reasoning items	Different materials items	Different materials items	Pulling items	Pulling items
	Model 1 (*n* = 144)	Model 2 (n = 136)	Model 3 (*n* = 144)	Model 4 (n = 136)	Model 5 (*n* = 144)	Model 6 (n = 136)
Variable	*β* (SE)	*β* (SE)	*β* (SE)	*β* (SE)	*β* (SE)	*β* (SE)
MRT	.22 (.08)**	.24 (.09)**	.15 (.09)	.18 (.10)	.26 (.09)**	.29 (.10)**
PFT	.31 (.09)***	.34 (.09)***	.21 (.10)*	.21 (.10)*	.32 (.10)**	.30 (.10)**
BT	.07 (.08)	.06 (.09)	.24 (.09)**	.25 (.10)*	− .12 (.09)	− .11 (.10)
WS	.17 (.08)*	.16 (.08)*	.13 (.08)	.15 (.09)	− .09 (.08)	− .11 (.09)
Age		.04 (.03)		.03 (.03)		.00 (.03)
Gender		− .05 (.16)		− .21 (.18)		− .06 (.18)
*R*-squared	32.58	36.48	25.32	26.68	20.09	20.75
*F*-stat (*df*)	16.79*** (4, 139)	12.35*** (6, 129)	11.78*** (4, 139)	7.83*** (6, 129)	8.73*** (4, 139)	5.63*** (6, 129)

Only men and women were included in models 2, 4, and 6

*MRT* Mental rotation task—standardized, *PFT* paper folding task—standardized, *BT* bending task—standardized, *KRT* knot reasoning task—arcsine-transformed and standardized, *WS* Wordsum Plus—standardized.

##### Relations among gender, spatial skills, and spatial activities engagement

**Fig. 3:**
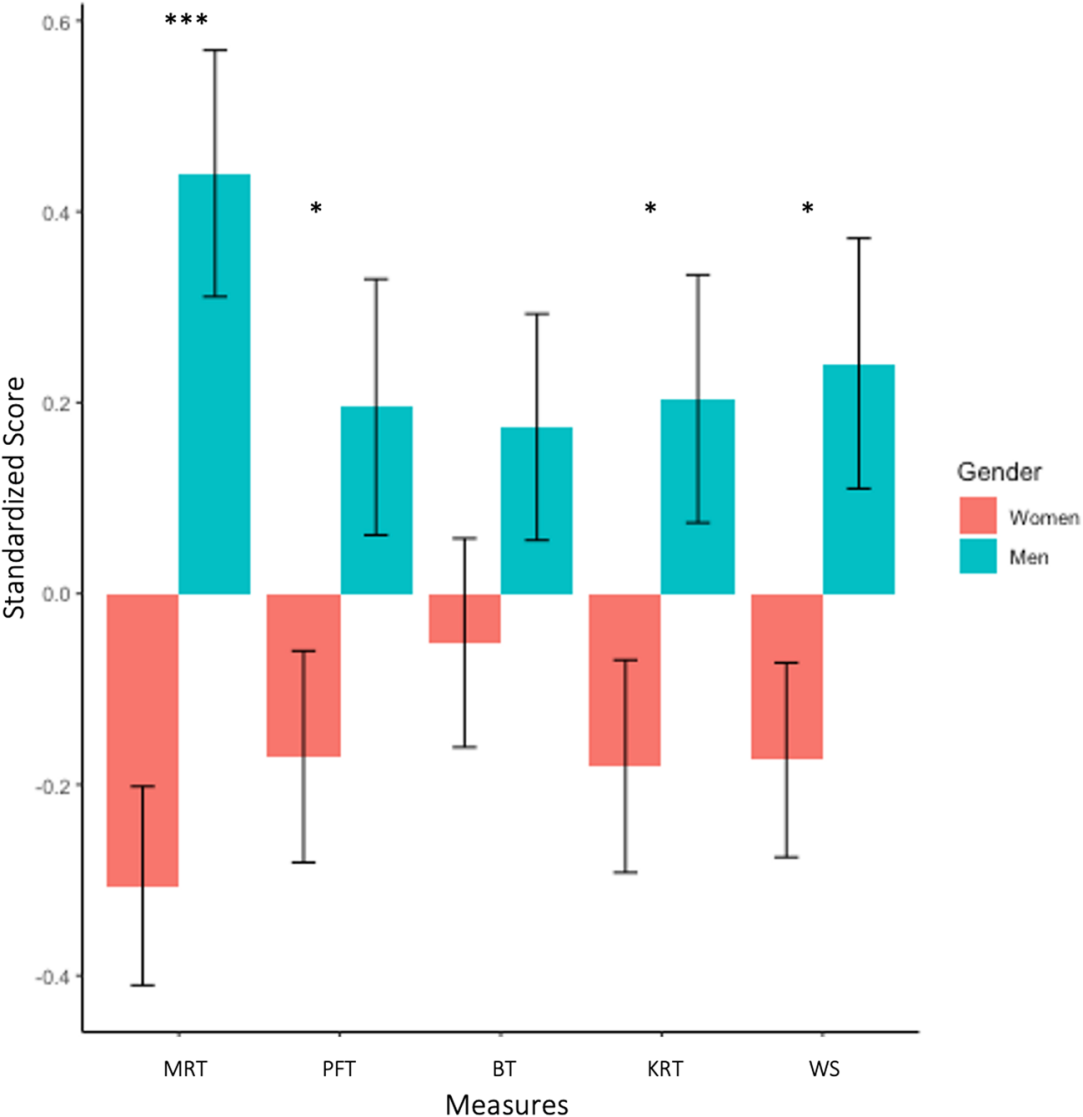
Performance on cognitive measures by gender—men and women. *Note.* Stars represent *p* values from Benjamini–Hochberg correction, *p < .05, **p < .01, ***p < .001. *MRT* Mental rotation task, *PFT* paper folding task, *BT* bending task, *KRT* knot reasoning task, *WS* Wordsum Plus task

^12^

To test whether gender related to spatial skills performance and engagement in spatial activities, we conducted a Welch’s between-groups *t*-test for each cognitive measure comparing men and women’s performance with a Benjamini Hochberg correction for five comparisons. Consistent with prior research (e.g., Lauer et al., 2019) and our predictions, we found that men outperformed women on the mental rotation task (*t*(116.39) = − 4.50, corrected *p* < 0.001, *d* = 0.78). Unexpectedly, men performed significantly better on the Wordsum Plus and paper folding tasks than women after correction for multiple comparisons (Wordsum: *t*(113.15) = − 2.50, corrected *p* = 0.034, *d* = 0.44; paper folding: *t*(118.32) = − 2.11, corrected *p* = 0.047, *d* = 0.36).^11^ We did not have a priori predictions about gendered patterns of performance on the bending and knot reasoning tasks, because performance on these tasks by gender had not been tested previously. There was no gender difference in performance on the bending task (*t*(124.77) = − 1.40, corrected *p* = 0.164, *d* = 0.24) but men outperformed women on the knot reasoning task (*t*(121.15) = − 2.25, corrected *p* = 0.044, *d* = 0.39) (Fig. 3).

**Fig. 4:**
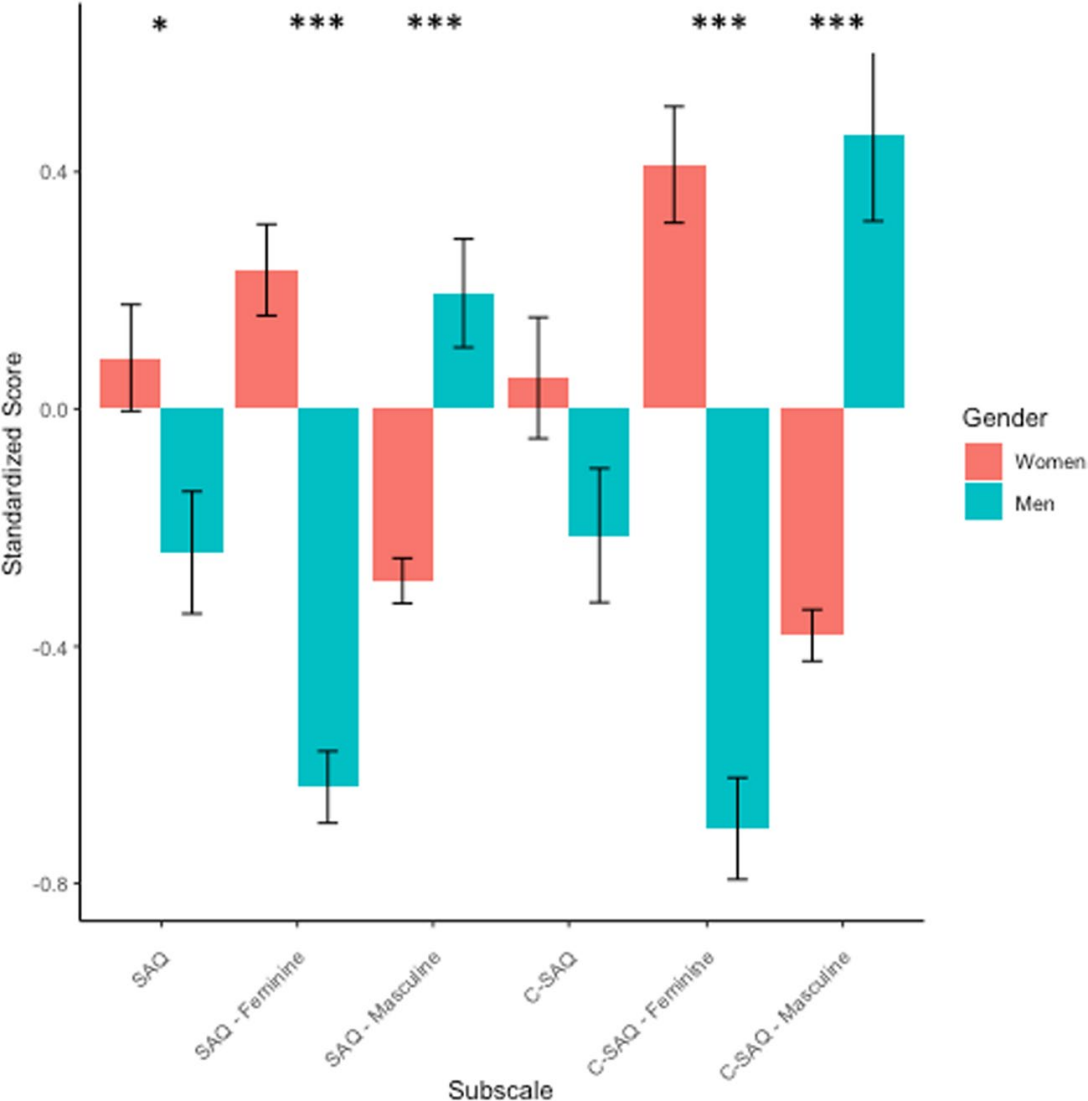
Responses to current and childhood spatial activities by gender—men and women. *Note.* Stars represent p-values from Benjamini–Hochberg correction, *p < .05, **p < .01, ***p < .001. *SAQ* Spatial Activities Questionnaire, *SAQ masculine* Spatial Activities Questionnaire Masculine-Stereotyped Items, *SAQ feminine* Spatial Activities Questionnaire Masculine-Stereotyped Items, *C-SAQ* Childhood Spatial Activities Questionnaire, *C-SAQ masculine* Childhood Spatial Activities Questionnaire Masculine-Stereotyped Items, *C-SAQ feminine* Childhood Spatial Activities Questionnaire Feminine-Stereotyped Items

Next, we examined gender differences and similarities in current and childhood spatial activities engagement, again using a Benjamini–Hochberg correction for 6 comparisons. Women reported significantly greater overall current spatial activities engagement (*t*(120.94) = 2.40, corrected *p* = 0.021, *d* = − 0.41), but there was not a significant difference for childhood spatial activities (*t*(123.57) = 1.74, corrected *p* = 0.084, *d* = − 0.30). In alignment with prior work (Doyle et al., 2012; Nazareth et al., 2013) and our prediction, men reported greater participation in masculine-stereotyped activities than women currently (*t*(70.04) = − 4.90, corrected *p* < 0.001, *d* = 0.98) and in childhood (*t*(62.36) = − 5.52, corrected *p* < 0.001, *d* = 1.13). Also consistent with our predictions, women reported greater participation in feminine-stereotyped activities than men currently (*t*(132.69) = 8.90, corrected *p* < 0.001, *d* = − 1.45) and in childhood (*t*(135.52) = 8.59, corrected *p* < 0.001, *d* = − 1.40) (Fig. 4).

##### Does spatial activities engagement relate to spatial skills and mediate gender differences in spatial skills?

**Fig. 5:**
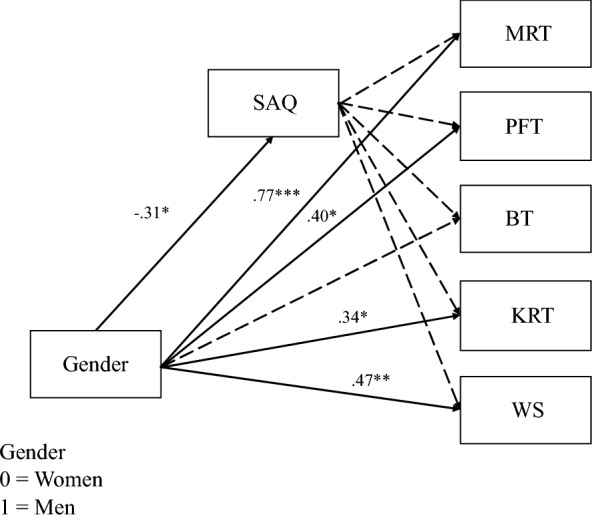
Structural equation model with gender and overall SAQ predicting cognitive measures. *Note.* *MRT* Mental rotation task—standardized, *PFT* paper folding task—standardized, *BT* bending task—standardized, *KRT* knot reasoning task—arcsine-transformed and standardized, *WS* Wordsum Plus—standardized. Solid lines indicate significant paths, and dashed lines indicate nonsignificant paths. Significant paths are labeled with the path coefficients. *p < .05, **p < .01, ***p < .001

To examine whether spatial activities engagement relates to spatial skills and mediates gender differences in spatial skills, we ran a structural equation model using the R package lavaan with gender and overall SAQ score predicting performance on all five cognitive skills measures (Fig. 5). This model only included participants who identified as men or women. Gender predicted overall SAQ (*β* = − 0.32, SE 0.14, *p* = 0.022), such that women reported higher overall SAQ than men. We hypothesized that greater current engagement in spatial activities (overall) would be positively associated with paper folding performance (replicating Peterson et al., 2020); however, this was not the case (*β* = 0.05, SE 0.11, *p* = 0.625). Overall SAQ scores were also not associated with bending performance (*β* = − 0.05, SE 0.10, *p* = 0.634) or knot reasoning performance (*β* = − 0.13, SE 0.11, *p* = 0.239), relationships that had not been tested previously.

Gender predicted participants’ performance on the mental rotation task (*β* = 0.77, SE 0.17, *p* < 0.001), the paper folding task (*β* = 0.40, SE 0.18, *p* = 0.022), the knot reasoning task (*β* = 0.34, SE 0.17, *p* = 0.047), and the Wordsum Plus task (*β* = 0.47, SE 0.16, *p* = 0.004); men outperformed women on these tasks, while accounting for current spatial activities. We next ran three structural equation models with the backwards reasoning, different materials, and pulling items modeled separately. The backwards reasoning items showed the same pattern of results as the overall knot reasoning task. However, gender no longer predicted performance on the different materials and pulling subscales of the knot reasoning task while accounting for current spatial activities (different materials: *β* = 0.19, SE 0.17, *p* = 0.271, pulling: *β* = 0.17, SE 0.18, *p* = 0.333; Appendix D, Figs. 17, 18 and 19).

**Fig. 6:**
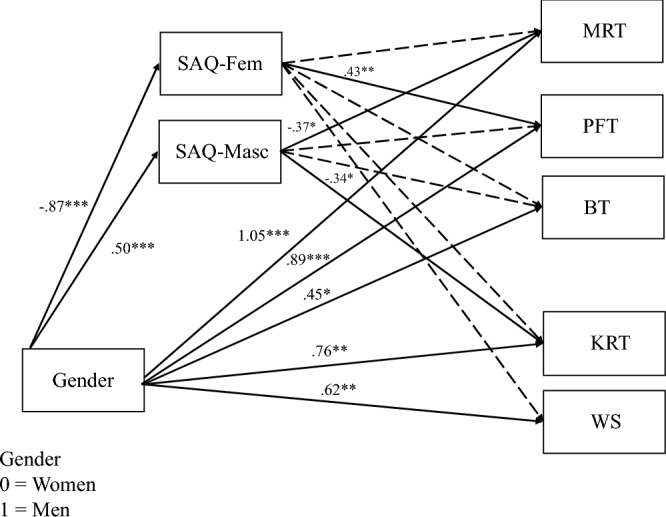
Structural equation model with gender and gendered SAQ predicting cognitive measures. *Note.* *MRT* Mental rotation task—standardized, *PFT* paper folding task—standardized, *BT* bending task—standardized, *KRT* knot reasoning task—arcsine-transformed and standardized, *WS* Wordsum Plus—standardized. Solid lines indicate significant paths, and dashed lines indicate nonsignificant paths. Significant paths are labeled with the path coefficients. *p < .05, **p < .01, ***p < .001

Next, we explored the relationship among current gender-stereotyped spatial activities, gender, and the cognitive tasks by running a structural equation model with gender, current expanded feminine-stereotyped spatial activities, and current masculine-stereotyped spatial activities predicting the cognitive skills measures (Fig. 6). Again, men outperformed women on all cognitive tasks (0.001 < *p* < 0.046). We hypothesized that greater current engagement in masculine-stereotyped spatial activities, but not feminine-stereotyped spatial activities, would be positively associated with mental rotation performance (replicating Nazareth et al., 2013). However, masculine-stereotyped spatial activities were significantly, *negatively* associated with mental rotation performance, while accounting for participant gender (*β* = − 0.37, SE 0.16, *p* = 0.023). Men showed better performance on the mental rotation task than women (*β* = 1.05, SE 0.22, *p* < 0.001), so we further hypothesized that the relationship between gender and mental rotation performance would be mediated by current masculine-stereotyped spatial activity engagement (consistent with Nazareth et al., 2013). We tested the indirect effect using bias-corrected bootstrap estimation with 1,000 iterations and considered effects to be significant if the 95% confidence interval did not include zero. We found a significant indirect effect, but again in the opposite direction than expected (*β* = − 0.19, 95% CI  [− 0.35, − 0.05]).

Second, we hypothesized that current engagement in the expanded feminine-stereotyped activities would predict participants’ performance on the paper folding, bending and knot reasoning tasks, but not their performance on the rigid mental rotation task. Feminine-stereotyped activities predicted performance on the paper folding task alone, accounting for participant gender (*β* = 0.43, SE 0.14, *p* = 0.002), and there was a significant indirect effect from gender to feminine-stereotyped activities to paper folding (*β* = − 0.37, 95% CI  [− 0.66, − 0.11]). Surprisingly, masculine-stereotyped activities predicted performance on the knot reasoning task—higher endorsement of masculine-stereotyped activities was associated with lower knot reasoning task performance (*β* = − 0.34, SE 0.17, *p* = 0.047) and there was a significant indirect effect from gender to masculine-stereotyped activities to knot reasoning task performance (*β* = − 0.17, 95% CI  [− 0.36, − 0.03]).

When we ran models with each type of knot reasoning item separately, feminine-stereotyped activities predicted performance on both the paper folding task *and* the backwards reasoning items (*β* = 0.31, SE 0.13, *p* = 0.020). Further, there was a significant indirect effect from gender to feminine-stereotyped activities to backwards reasoning performance, meaning that women who did more feminine-stereotyped activities did better on the backwards reasoning items (*β* = − 0.27, 95% CI  [− 0.52, − 0.06], Appendix D, Fig. 20). The patterns for the different materials and pulling items were similar to the model with overall knot reasoning task performance, but there was no longer a significant direct effect of gender on pulling task performance (Appendix D, Figs. 21 and 22).

**Fig. 7:**
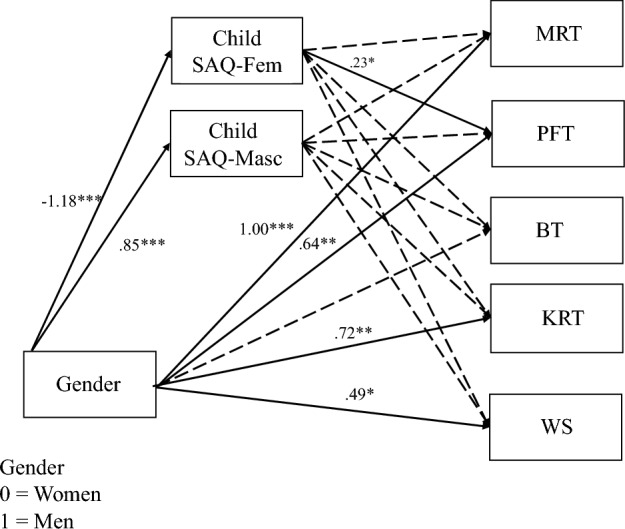
Structural equation model with gender and childhood gendered SAQ predicting cognitive measures. *Note.* *MRT* Mental rotation task—standardized, *PFT* paper folding task—standardized, *BT* bending task—standardized, *KRT* knot reasoning task—arcsine-transformed and standardized, *WS* Wordsum Plus—standardized. Solid lines indicate significant paths, and dashed lines indicate nonsignificant paths. Significant paths are labeled with the path coefficients. *p < .05, **p < .01, ***p < .001

Finally, we ran a model with gender, childhood expanded feminine-stereotyped spatial activities, and childhood masculine-stereotyped spatial activities predicting the cognitive skills measures (Fig. 7). We did not have an a priori hypothesis about the role of childhood spatial activities participation in participants’ spatial skills performance. Accounting for gender, childhood feminine-stereotyped spatial activities significantly predicted performance on the paper folding task (*β* = 0.23, SE 0.11, *p* = 0.039; Fig. 7). The indirect effect from gender to childhood feminine-stereotyped spatial activities to paper folding task performance was also significant (*β* = − 0.27, 95% CI  [− 0.54, − 0.01]. We found the same pattern when modeling each type of knot reasoning item separately, except there was no longer a significant effect of gender on the different materials and pulling subscales (Appendix D, Figs. 23, 24 and 25).

##### Do spatial skills differ by college major?

**Fig. 8:**
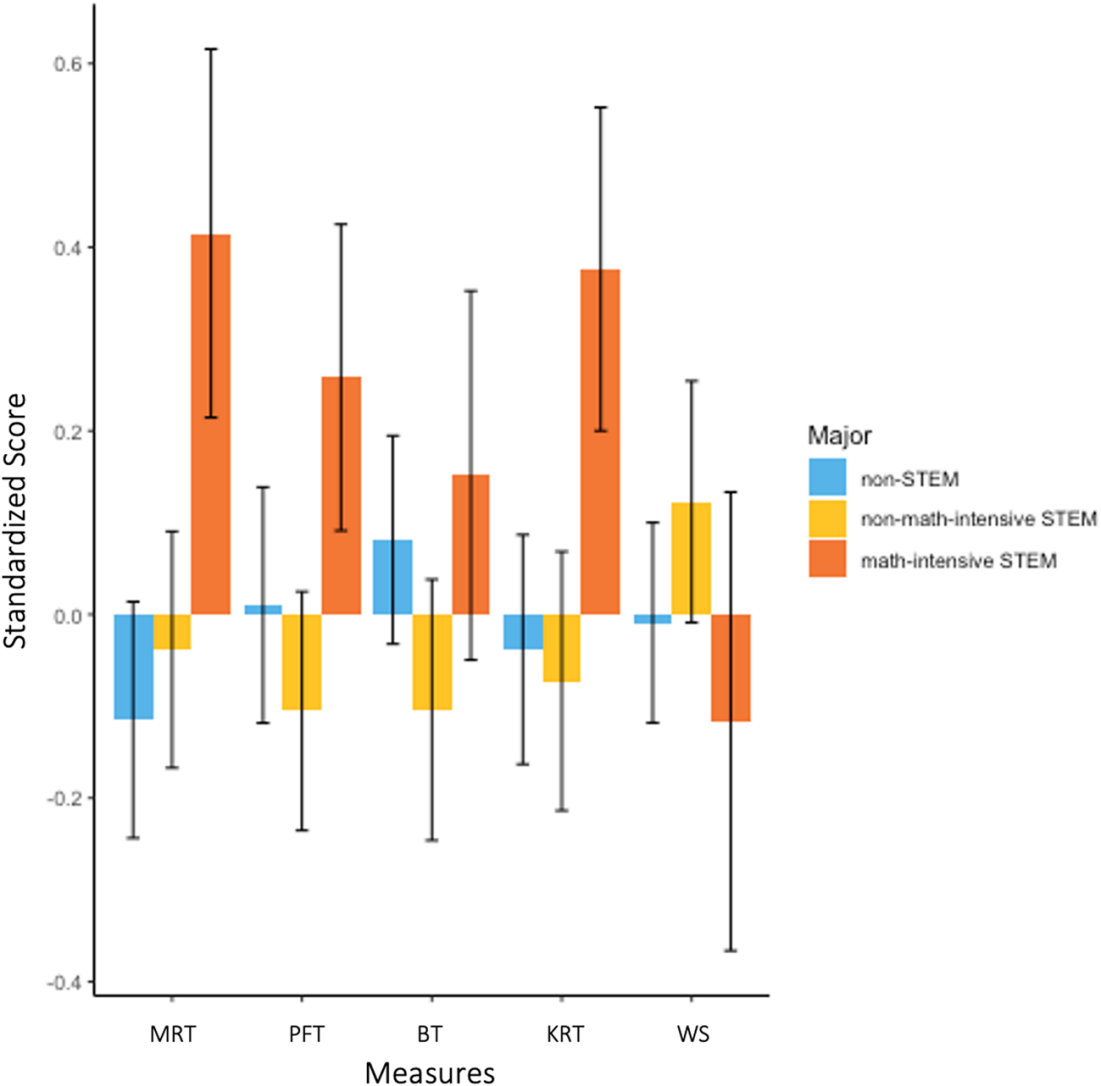
Performance on cognitive measures by major type. *Note.* *MRT* Mental rotation task, *PFT* paper folding task, *BT* bending task, *KRT* knot reasoning task, *WS* Wordsum Plus

**Fig. 17:**
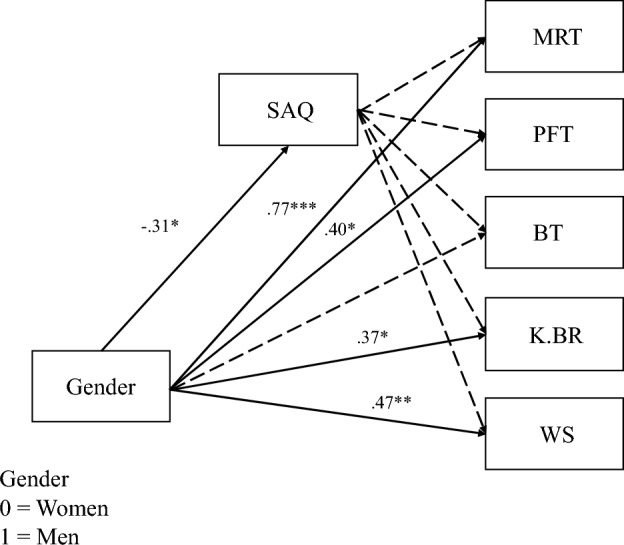
Structural equation model with gender and SAQ predicting cognitive measures—backwards reasoning items separately

**Fig. 18:**
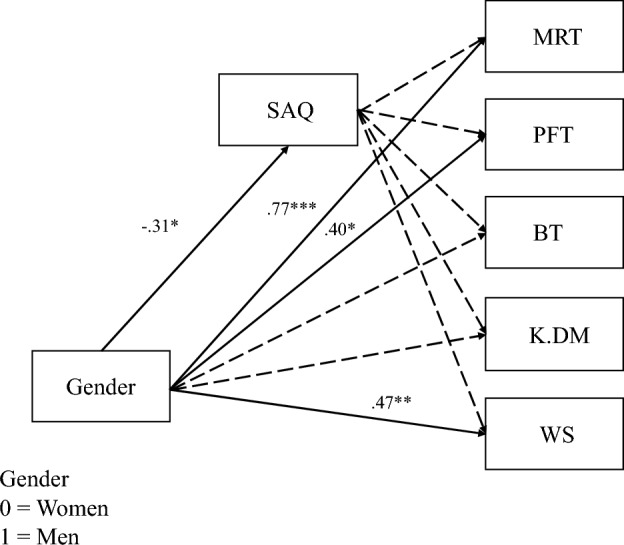
Structural equation model with gender and SAQ predicting cognitive measures—different materials items separately

**Fig. 19:**
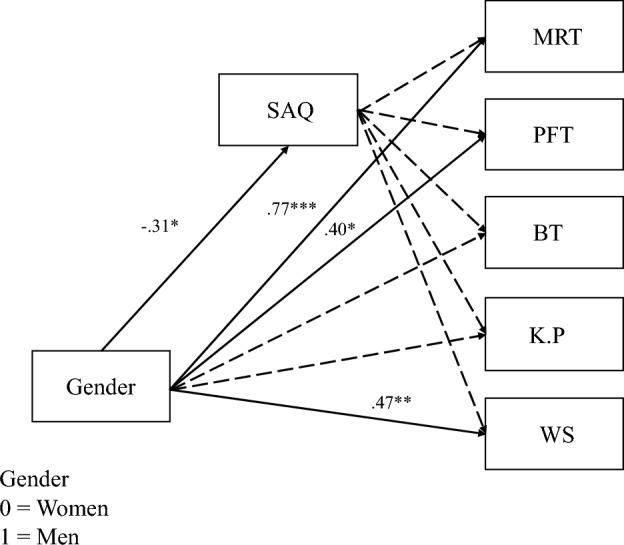
Structural equation model with gender and SAQ predicting cognitive measures—pulling items separately

**Fig. 20:**
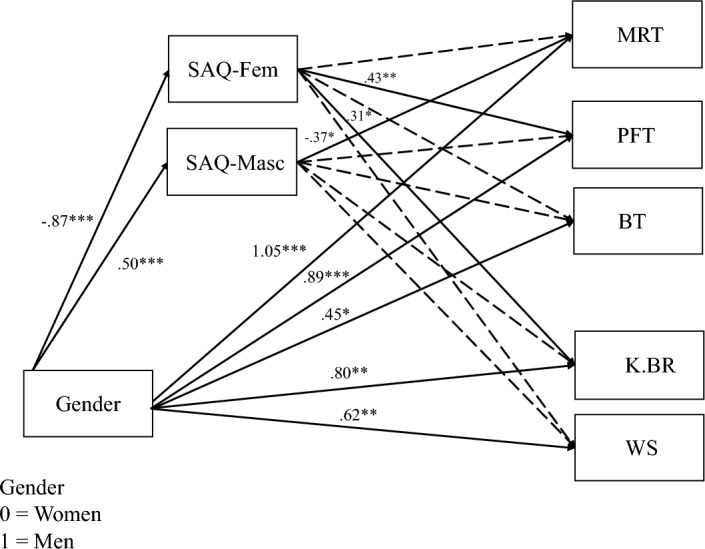
Structural equation model with gender and gendered SAQ predicting cognitive measures—backwards reasoning items separately

**Fig. 21:**
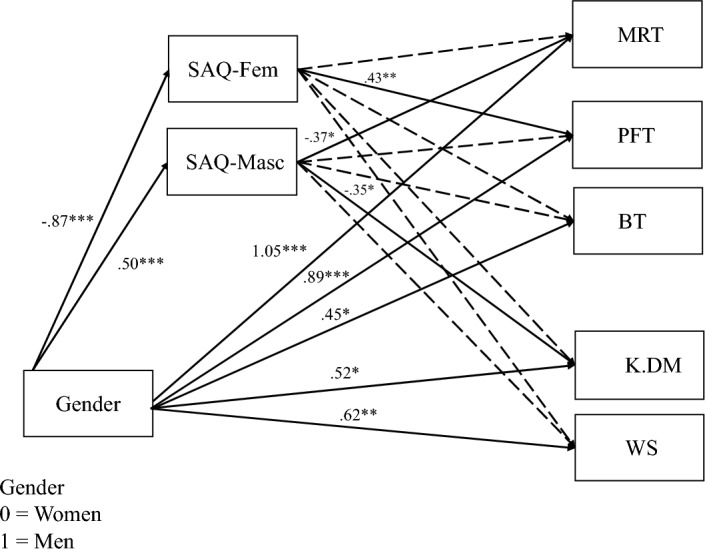
Structural equation model with gender and gendered SAQ predicting cognitive measures—different materials items separately

**Fig. 22:**
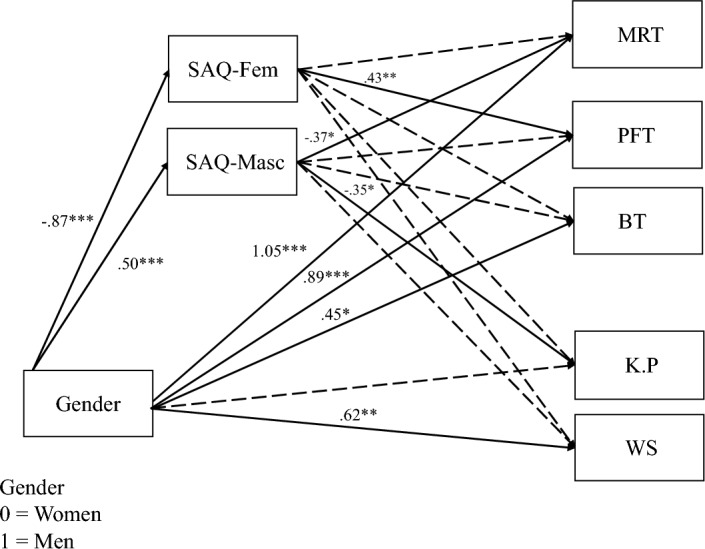
Structural equation model with gender and gendered SAQ predicting cognitive measures—pulling items separately

**Fig. 23:**
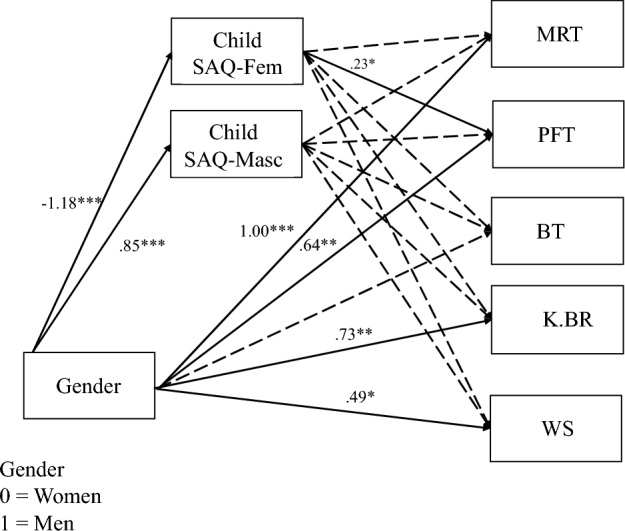
Structural equation model with gender and childhood gendered SAQ predicting cognitive measures—backwards reasoning items separately

**Fig. 24:**
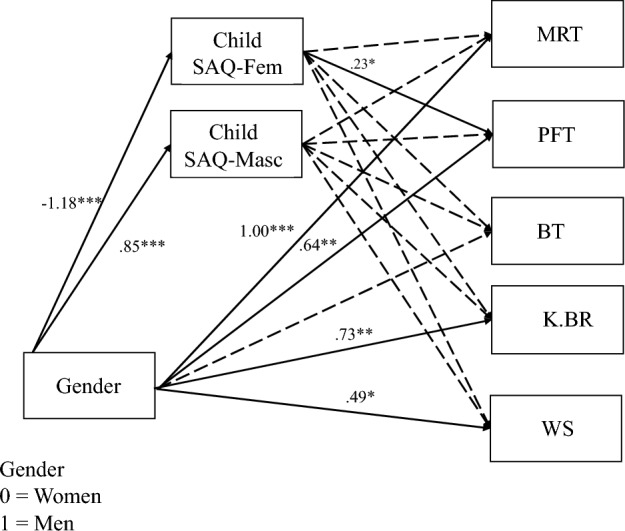
Structural equation model with gender and childhood gendered SAQ predicting cognitive measures—different materials items separately

**Fig. 25:**
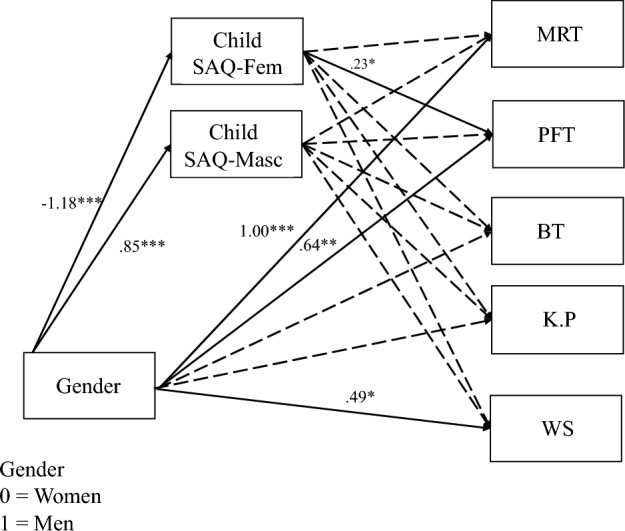
Structural equation model with gender and childhood gendered SAQ predicting cognitive measures—pulling items separately

Given prior work showing gender differences in STEM college major choice (Tian et al., 2023), we checked the percentage of men and women who reported being STEM majors as undergraduates: 61% of men and 51% of women were STEM majors, which was not a statistically significant difference (*t*(115.56) = − 1.13, *p* = 0.262, *d* = 0.20). In our study, unlike prior work, the percentage of men and women pursuing math-intensive STEM majors was also similar (*n* = 9 men, 27% of men; *n* = 12 women, 29% of women).^13^ Next, we looked descriptively at performance on each cognitive task by major type (Table 6). Finally, we ran a MANOVA with major as a categorical variable (math-intensive STEM, non-math-intensive STEM, and non-STEM) predicting performance on the four spatial measures, and found that contrary to our prediction, performance did not significantly differ by major (*p*s > 0.05; Fig. 8).

**Table 6:** Descriptive statistics for cognitive scores by major type

	All participants (*n* = 147)	Non-STEM majors (*n* = 64)	Non-math-intensive STEM majors (*n* = 56)	Math-intensive STEM majors (*n* = 23)
Variables	*M* (SD)	*M* (SD)	*M* (SD)	*M* (SD)
MRT	19.31 (11.05)	18.03 (11.34)	18.88 (10.62)	23.87 (10.60)
PFT	10.86 (3.97)	10.93 (4.07)	10.47 (3.85)	11.91 (3.10)
BT	.73 (.13)	.74 (.11)	.72 (.13)	.75 (.12)
KRT	.84 (.10)	.84 (.11)	.83 (.10)	.88 (.07)
WS	.68 (.15)	.67 (.13)	.69 (.15)	.66 (.18)

*MRT* Mental rotation task, *PFT* paper folding task, *BT* bending task, *KRT* knot reasoning task, *WS* Wordsum Plus

## Discussion

Across two studies, we created and validated a measure of non-rigid, ductile spatial skills based on knot reasoning. As we predicted, performance on the knot reasoning measure was significantly, positively correlated with existing measures of spatial skill (mental rotation, paper folding, bending). However, contrary to our predictions, we did not find support for a straightforward continuum of non-rigid to rigid spatial skills. Exploratory analyses suggest that the different item types in the knot reasoning task might function differently. Using two analytic approaches, we replicated prior work showing that men outperformed women on the mental rotation task, and, somewhat surprisingly, found the same pattern for the other spatial skills except bending. We also examined whether spatial activities engagement would mediate the relationship between gender and spatial skills performance. Unlike prior work, we found an inconsistent mediation effect, with men who reported greater engagement in masculine-stereotyped spatial activities having lower mental rotation and knot reasoning task performance. Engagement in feminine-stereotyped spatial activities mediated the relationship between gender and paper folding performance, with women who reported doing more feminine-stereotyped spatial activities having higher paper folding performance. While we did not find a similar indirect effect when examining the knot reasoning task as a whole, exploratory analyses showed that feminine-stereotyped spatial activities mediated the relationship between gender and performance on the backwards reasoning items specifically. Although math-intensive majors had higher mean scores on the spatial skills measures than other majors, spatial skills did not significantly differ among math-intensive STEM, non-math-intensive STEM, and non-STEM majors.

### Understanding the structure and variety of spatial skills

The current studies expand our toolkit for measuring spatial skills to include non-rigid, ductile transformations in non-planar materials. We replicated Atit et al.’s (2013) finding that paper folding but not mental rotation predicted bending task performance in a simultaneous regression. Extending on Atit et al.’s (2013) work, we first found that the knot reasoning task, like the bending task, was also significantly, positively correlated with existing spatial skills measures. We expected bending would be most closely related to knot reasoning because both tasks require thinking about non-rigid, ductile transformations. However, we found that the mental rotation (a rigid spatial skill) and paper folding tasks (a brittle, non-rigid spatial skill) but *not* the bending task (another non-rigid, ductile spatial skill) predicted performance on the knot task in a simultaneous regression. There are several possible explanations for these patterns. First, bending is an intermediate step of folding, which could explain an overlap between bending and folding but not an overlap between bending and knot reasoning. Second, the paper folding and bending tasks share surface and task features: Both involve imagining the transformation of a flat, planar object (a square piece of paper and a rectangular plastic sheet) and the movement of one or two points (holes, a star and circle) on the object. One future direction could be to test the bending task with another already existing (e.g., the DAT) or new measure of folding that shares fewer task features. Conversely, future work could create a knot reasoning measure with task structure aligned to the paper folding and bending tasks (e.g., identifying the left–right position between two points on a string when it is knotted versus unknotted). A third possible explanation lies in the static-to-dynamic dimension proposed by Newcombe and Shipley (2015). When we ran three separate regressions, with each subscale of the knot reasoning task predicted by mental rotation, paper folding, and bending, we found that the different materials subscale was predicted by paper folding and bending (but not mental rotation), as we initially hypothesized. However, the backwards reasoning and pulling subscales showed the same pattern as the overall knot reasoning task: They were predicted by mental rotation and paper folding but not bending. We suggest that the backwards reasoning and pulling subscales of the knot reasoning task may rely on the same dynamic reasoning that the mental rotation and paper folding tasks do. The different materials subscale, on the other hand, could be solved with a pattern-matching strategy, which could explain why the mental rotation task does not predict performance on this subscale. In other words, we must consider the static-to-dynamic continuum in addition to the rigid-to-non-rigid continuum when assessing the relationships among spatial skills. Creating and testing additional measures of continuous planar and string measures would allow us to further test these conceptual dimensions, using, for example, a confirmatory factor analytic approach (CFA). A current lack of measurement tools—particularly for non-rigid spatial skills—hampers our ability to move toward a comprehensive understanding of spatial thinking: Without an adequate number of measures, our factor analytic approaches will be under-identified (as pointed out by Hegarty & Waller, 2005).

### Gender and spatial skills

Turning to gender differences and similarities in spatial skills, we partially replicated prior work. We conducted both pairwise contrasts and structural equation modeling to test whether men and women differed in their cognitive task performance. We replicated prior work (e.g., Lauer et al., 2019), showing that men outperformed women on the mental rotation task. However, contrary to our predictions and prior research (e.g., Harris et al., 2013), we found that men outperformed women on the paper folding task. We also report a novel finding that there were generally not gender differences in performance on the bending task in either analysis approach, and that men outperformed women on the knot reasoning task. Again, we were surprised by the significant gender difference in non-rigid spatial skill performance given prior work on paper folding and a preexisting hypothesis that the rigid versus non-rigid distinction could be an organizing factor for gender differences in spatial skill (e.g., Harris et al., 2013). It is important to note that men in our study also outperformed women on our verbal measure, with an effect size similar to the male advantage in the bending and knot reasoning tasks, in contrast to prior studies showing either no gender difference or a female advantage in verbal skills (e.g., Hyde & Linn, 1988; Lietz, 2006). This suggests that men in our sample may have been generally higher-achieving than women across domains. Therefore, we are cautious about over-interpreting gender differences in performance on the non-rigid spatial skills measures, especially given the large body of prior work that does not show differences in the paper folding task. Future work should try to replicate the gendered patterns of performance that we found in the non-rigid spatial tasks.

### Gender and spatial activities experience

Consistent with prior research and our predictions, masculine- and feminine-stereotyped spatial activities showed greater endorsement by men and women, respectively (Munns et al., 2022; Newcombe et al., 1983; Peterson et al., 2020; Signorella et al., 1986). Given possible changes in activity popularity over time, recent work has both investigated current frequencies of the original activities included in the Spatial Activities Questionnaire and added navigational and computer-related activities (Munns et al., 2022). In our sample, we found a somewhat lower range of frequency in current spatial activities than Munns et al., 2022 (present study: 1.01–2.77, where 1 indicated never engaging in the activity and 6 indicated engaging in the activity more than once a week; Munns et al. (2022): 1.05–4.79). Notably, in our study, participants did report engaging in the twelve additional fiber arts activities (item means: 1.01 to 2.77, all 12 items above 1), suggesting that these activities reflect meaningful variability in spatial exposure.

### The interplay among gender, gendered spatial activities experience, and spatial skills

Once we considered gender and spatial activities jointly, we found novel relationships among gender, gender-stereotyped spatial activities, and spatial skills. First, we found that women reported engaging in more spatial activities overall than men, contrary to our prediction that there would be no difference. However, we added items to the feminine-stereotyped subscale and women reported engaging in significantly more feminine-stereotyped activities than men. When we replicated our analysis with only the original 30 spatial activities, which had an equal number of feminine- and masculine-stereotyped spatial skills, the gender difference in overall spatial activities engagement went away.

More interestingly, we found that current engagement in masculine-stereotyped spatial activities mediated the relationship between gender and mental rotation performance. Although prior work has found this indirect effect (Nazareth et al., 2013), in our sample it went in the opposite direction: Men who reported engaging in *more* masculine-stereotyped spatial activities had *lower* mental rotation task scores. At the same time, men tended to do better on the mental rotation task than women and tended to report engaging in more masculine-stereotyped spatial activities than women, consistent with prior work. Thus, in our study, masculine-stereotyped spatial activities exerted a suppression effect on the relation between being a man and having higher mental rotation performance (also referred to as inconsistent mediation). One possible explanation is that the tasks included in the masculine-stereotyped activities subscale reflect normative activities from a different time period, and that men’s current advantage in mental rotation could be driven by a different set of masculine-stereotyped spatial activities that are now more common and more predictive of spatial skill. For example, a recent study showed that men endorsed engaging in computer games at high frequency (Munns et al., 2022). One complementary possibility is that newer activities (e.g., video games) have also displaced time once spent on the activities measured in the original spatial activities scale (e.g., football). While a change in masculine-stereotyped spatial activities might explain a lack of a mediation effect among gender, masculine-stereotyped spatial activities, and mental rotation task performance, it does not entirely explain the inconsistent mediation. More research is needed to understand potential historical changes in the frequency of specific masculine-stereotyped activities and their relations to spatial skills.

We also found two novel significant indirect effects. First, contrary to our predictions, masculine-stereotyped current spatial activities mediated the relationship between gender and knot reasoning task score. Similar to the mental rotation task, men who reported engaging in more masculine-stereotyped spatial activities had *lower* knot reasoning task performance. We are not certain why this inconsistent mediation exists, but could be aligned with the unexpected relationship we found between the mental rotation and knot reasoning tasks.

Second, consistent with our predictions, feminine-stereotyped current and childhood spatial activities mediated the relationship between gender and paper folding task score. Women who reported engaging in more feminine-stereotyped spatial activities had higher paper folding performance. Further, when we modeled each subscale of the knot reasoning task separately, we found a similar mediation for the backwards reasoning items. Notably, the relationships between feminine-stereotyped activities and paper folding and the backwards reasoning items were the only significant, positive relationships between activities and skills. The majority of the activities in the feminine-stereotyped subscale were fiber arts, though the paper folding mediation still held when we examined only the original 10 items. Newcombe et al. (1983) also found that several fiber arts activities were significantly, positively correlated with performance on another measure of paper folding (DAT). Although most prior work has focused on the role of masculine-stereotyped spatial activities on spatial skill, our findings suggest that future work should investigate feminine-stereotyped spatial activities—specifically fiber arts—as avenues for improving spatial skill.

### STEM major choice and spatial skills

Finally, we did not find that major was significantly related to performance on the spatial skills measures, contrary to our expectations and prior work (Tian et al., 2023). However, we had a relatively small number of math-intensive STEM majors in our sample (*n* = 23). Directionally, math-intensive STEM majors did have higher performance on the four spatial skills, especially the mental rotation task. It is possible that in a larger sample with a more even distribution of major types, significant differences in spatial skills performance would emerge.

One intriguing future direction would be to examine the relation between specific spatial skills and STEM majors categorized at a more fine-grained level. Other researchers have proposed that non-rigid spatial skills could be uniquely important for STEM fields focused on non-rigid phenomena, such as geology, atmospheric sciences, and oceanography (e.g., McNeal & Petcovic, 2020). Future work could test whether students in STEM fields focused on non-rigid phenomena have better non-rigid spatial skills than students in other STEM fields. Another approach would be to compare STEM experts’ performance on an expanded suite of rigid and non-rigid spatial tasks, similar to Resnick and Shipley (2013). Although expert studies do not allow us to determine causality, it would be a useful first empirical investigation of the claim that some STEM (and potentially arts) fields rely on specifically non-rigid spatial skills.

### Limitations

Our conclusions are limited by several design and sample limitations. First, compared with existing spatial skills measures (the mental rotation task, paper folding task, and bending task), performance on the knot reasoning measure was relatively high, especially in the in-person sample for Study 2. Notably, the knot reasoning task was the only spatial skill measure that did not have a time limit. Given prior work showing that time limits can shift participants’ responses on the mental rotation task (Voyer et al., 1995), future work could add a time limit to better match the format of other spatial skills measures. Further, to decrease the length of the measure, future researchers could consider dropping some of the items with near ceiling performance. The IRT analyses suggested that the knot reasoning task may skew toward “easier” items, so future work could also explore adding more challenging items.

Second, we have identified the rigid versus non-rigid and static versus dynamic continua as important dimensions to explore going forward. Although the studies reported here help advance the investigation of these constructs, our design lacked a sufficient number of spatial skills measures to explicitly test the structure of spatial skills using a CFA approach.

Third, we recognize that our analyses of the role of gender in spatial skills and spatial activities exposure are limited to men and women. We actively recruited non-binary participants to help address the lack of research on spatial skills and gender outside of the binary. To our knowledge, transgender people (including non-binary people) are not well reflected in the current literature on gender and spatial skills, although researchers have studied transgender men’s spatial skills (e.g., Peragine et al., 2022). We planned to run inferential statistics with non-binary participants as a separate group if we had at least ten non-binary participants. Although we did not reach this threshold, we included non-binary participants in our other analyses, and reported descriptive statistics for non-binary participants as a group. We hope that this can serve as a first step toward future work that could focus more explicitly on transgender people’s spatial skills, including people who do not identify within the gender binary. Additionally, we did not try to disentangle gender and sex in either of our samples—we asked participants to report their gender identity only. Therefore, we did not differentiate between transgender and cisgender men and women. Building off of existing work on the role of gender and activational sex hormones on mental rotation skill (Peragine et al., 2022), future work could study both gender and sex to further understand the roles of hormones, environment, and socialization on spatial activities engagement and spatial skills.

Finally, our sample was US-based, and, in the case of Study 2, primarily undergraduate students. On the one hand, our sample is similar to many prior studies focused on spatial activities engagement in adults. The original Spatial Activities Questionnaire studies by Newcombe et al. (1983) and Signorella et al., (1986), as well as Nazareth et al. (2013), Munns et al. (2022) study, and the studies reported here all sampled undergraduate students in the USA. However, our findings and those of Munns et al. (2022) suggest that even within US college students there has been both change and consistency in gendered activity endorsement over a forty-year period. It is possible that endorsement of different spatial activities would vary if we sampled different age groups at different points in time, as well as sampled from non-college-educated, or non-US samples more generally. We suggest that attempts to incorporate additional spatial activities and examine their frequency in different time and cultural contexts could help future researchers better understand variation in spatial concept exposure and its potential impact on spatial skills.

## Conclusion

In conclusion, the present studies move us a step closer to having tools to measure the breadth of spatial skills. The knot reasoning task is a reliable and valid measure of adults’ non-rigid, ductile spatial reasoning. Our findings suggest that future work to develop measurement of non-rigid spatial skills in particular would allow us to better test the structure of spatial skills. Finally, we show initial evidence for the role of feminine-stereotyped spatial activities, including fiber arts activities, in explaining spatial skills performance.

### Open practices statement

Data and materials are available on Open Science Framework (OSF) at this link: https://osf.io/x9uqe/?view_only=84f43a20dcf848738e9f70f2caff17c1. Neither study reported here were preregistered.

## Figures and Tables

**Fig. 9:**
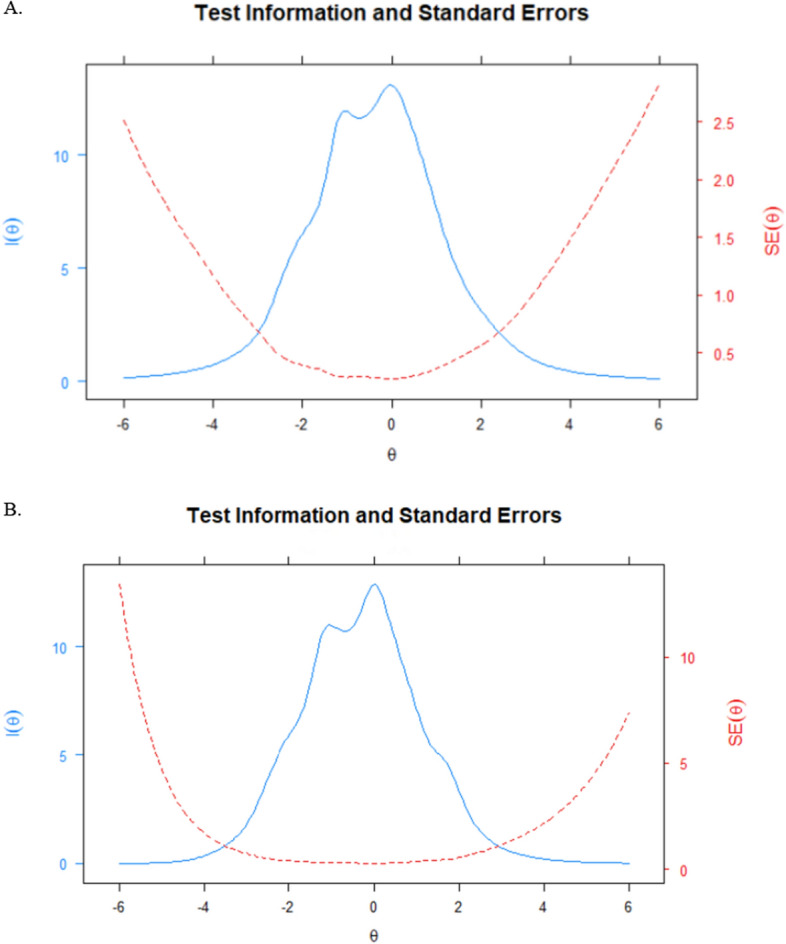
Study 1 standard error plots for 73-item and 54-item 2PL models—knot reasoning task. *Note.* This figure shows the standard error plots for **a** the 2PL model 73-item knot reasoning task from Study 1 and **b** the 2PL model 54-item knot reasoning task from Study 1

**Fig. 10:**
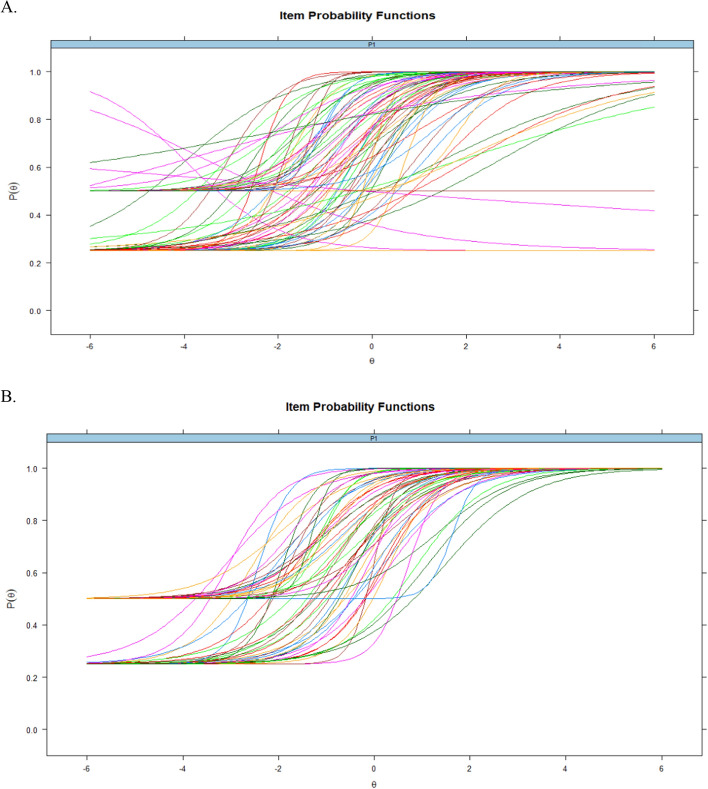
Study 1 IRT 2PL model item characteristic curves. *Note.* This figure shows the item characteristic curves for each item in the **a** full 73-item knot reasoning task from Study 1 and **b** shortened 54-item knot reasoning task from Study 1

**Fig. 11:**
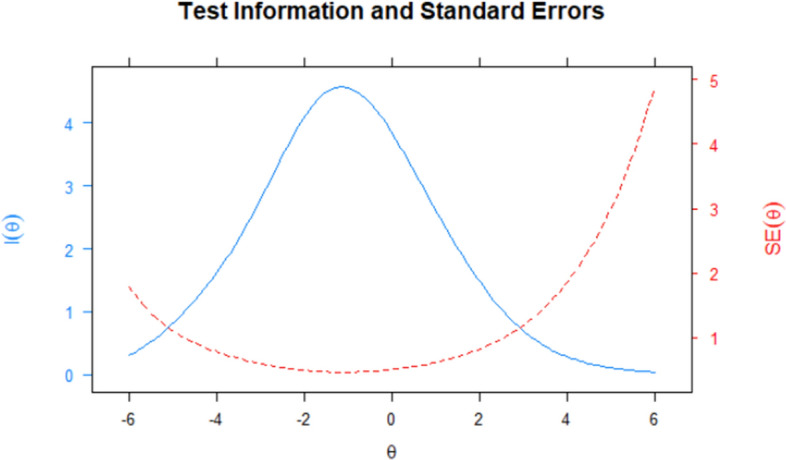
Study 2 1PL model standard error plot for knot reasoning task. *Note.* This figure shows the standard error plot for the 1PL model of the 54-item knot reasoning task from Study 2

**Fig. 12:**
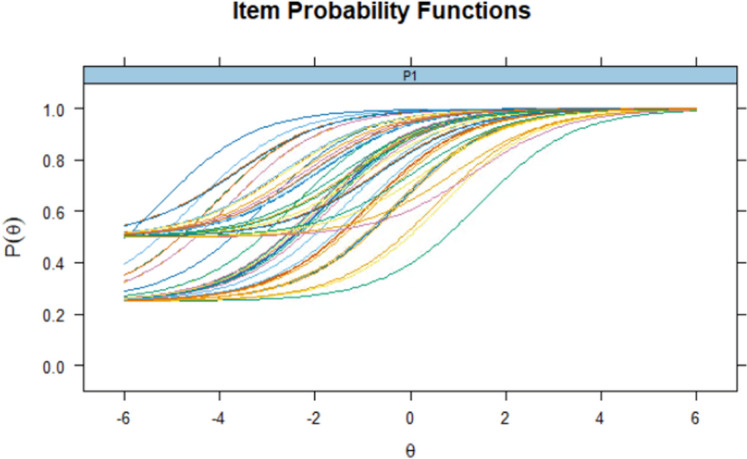
Study 2 item characteristic curves. *Note.* This figure shows the item characteristic curves for the 1PL model of the 54-item knot reasoning task from Study 2

**Fig. 13:**
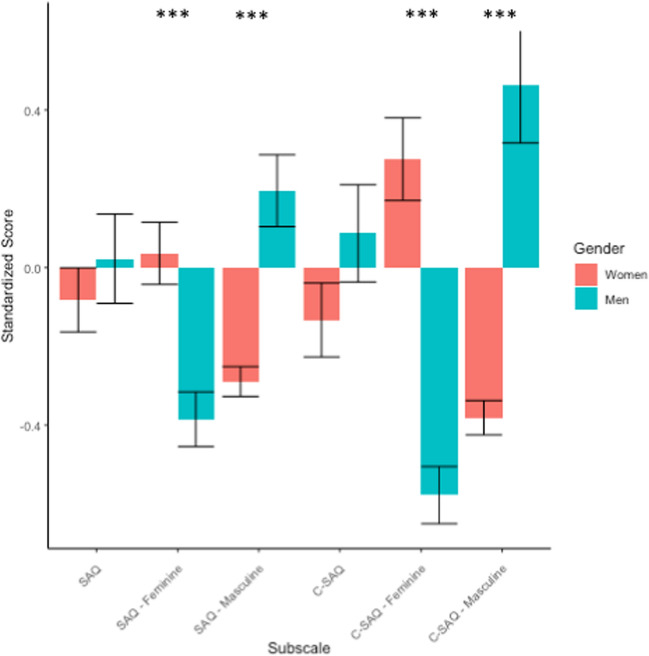
Responses to current and childhood spatial activities (30 items) by gender—men and women. *Note.* Stars represent *p* values from Benjamini–Hochberg correction, *p < .05, **p < .01, ***p < .001

**Fig. 14:**
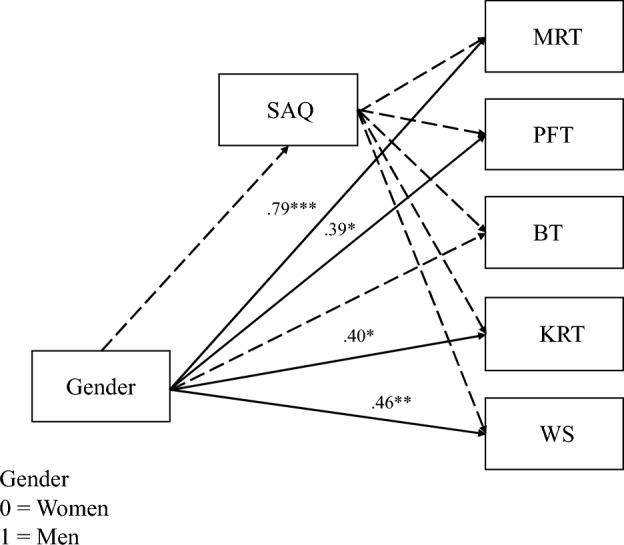
Structural equation model with gender and overall SAQ (30 items) predicting cognitive measures

**Fig. 15:**
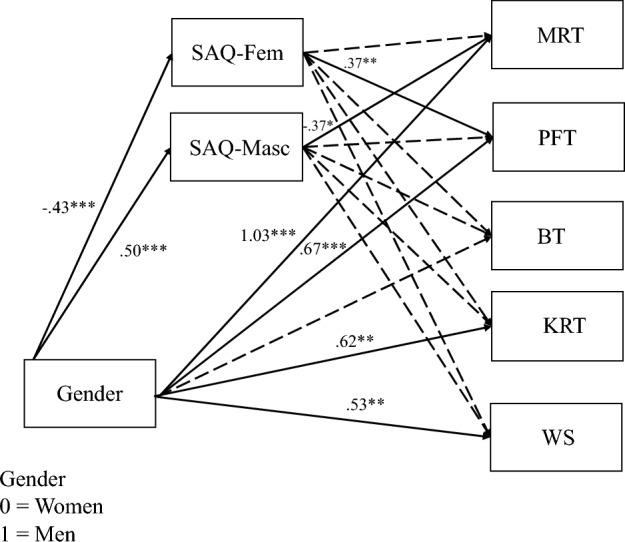
Structural equation model with gender and gendered SAQ (30 items) predicting cognitive measures

**Fig. 16:**
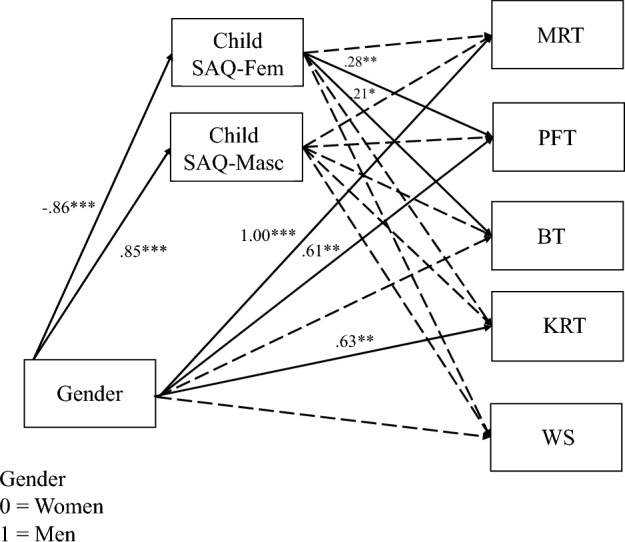
Structural equation model with gender and childhood gendered SAQ (30 items) predicting cognitive measures

## Appendix A: Instructions for knot reasoning task

### Backwards reasoning

“In this test you are to imagine the knotting and unknotting of rope. For each problem there is a target image (above) that shows a single step of a knot. The photographs below will be steps of knots that you will evaluate based on the instructions. You will indicate your answers by selecting the photograph that matches the instructions. Some of the images will be knots tied with one rope, others will be knots tied with two ropes. Some knots will be tied to an object, while others will be freestanding. For the following questions, indicate which photograph shows the step directly before the target photograph. I'll be seated over there doing some work. Please click >> to go to the next page.”

### Different materials

“In this test, you are to imagine the knotting and unknotting of rope. In each problem there is a target image (above) that shows a single knot. The photographs below will be knots made of different materials. You will indicate your answers by selecting the photograph that matches the instructions. Some of the images will be knots tied with one rope, others will be knots tied with two ropes. For the following questions, indicate which photograph below shows the same knot as the target photograph. I'll be seated over there doing some work. Please click >> to go to the next page.”

### Pulling

“In this test, you are to imagine the knotting and unknotting of rope. In each problem there is a target image that shows a single knot. One end of the rope will be circled and have an arrow next to it. For these questions, imagine pulling the circled end in the direction of the arrow. Indicate ‘yes’ if the knot will come apart or ‘no’ if the knot will not come apart. Some of the images will be knots tied with one rope, others will be knots tied with 2 ropes. Some knots will be tied to an object, while others will be freestanding. If there is another end to the rope, imagine that it is held in place. If there is another object, imagine it is stationary and does not move. You may see the same knot more than once with different circles and arrows. I'll be seated over there doing some work. Please click >> to go to the next page.”

## Appendix B: Item response theory tables and figures

See Tables 7, 8, 9 and Figs. 9, 10, 11, 12.

**Table 7:** Study 1: item response theory 2PL model—item-level information about knot reasoning task (full—73 items)

Item	Proportion correct	Standard deviation	Discrimination parameter (*a*)	Difficulty parameter (*b*)	Guessing parameter (*g*)
BR_1	.59	.49	1.31	.15	.25
BR_2	.74	.44	.97	− .76	.25
BR_3	.50	.50	.51	1.45	.25
BR_4	.43	.50	.61	2.03	.25
BR_5	.42	.49	2.98	.70	.25
BR_6	.79	.41	1.00	− 1.10	.25
BR_7	.73	.44	1.21	− .62	.25
BR_8	.60	.49	1.90	.15	.25
BR_9	.37	.48	− .51	− 3.47	.25
BR_10	.63	.48	1.22	− .05	.25
BR_11	.80	.40	1.41	− .93	.25
BR_12	.63	.48	3.76	− .03	.25
BR_13	.71	.45	1.77	− .39	.25
BR_14	.66	.47	1.93	− .14	.25
BR_15	.72	.45	1.93	− .39	.25
BR_16	.73	.44	1.42	− .57	.25
BR_17	.55	.50	1.99	.31	.25
BR_18	.71	.46	1.29	− .43	.25
BR_19	.74	.44	1.72	− .52	.25
BR_20	.52	.50	.33	1.79	.25
BR_21	.62	.49	1.51	.01	.25
BR_22	.57	.50	1.37	.32	.25
BR_23	.27	.44	− 1.03	− 3.97	.25
BR_24	.39	.49	.58	2.67	.25
BR_25	.42	.49	1.08	1.47	.25
BR_26	.58	.49	1.80	.21	.25
BR_27	.67	.47	1.73	− .25	.25
BR_28	.47	.50	1.34	.89	.25
DM_1	.13	.33	.37	29.07	.25
DM_2	.79	.41	1.53	− .87	.25
DM_3	.97	.17	.91	− 3.99	.25
DM_4	.98	.13	3.33	− 2.38	.25
DM_5	.48	.50	.48	1.78	.25
DM_6	.96	.19	1.14	− 3.13	.25
DM_7	.96	.19	2.98	− 1.88	.25
DM_8	.88	.32	1.34	− 1.64	.25
DM_9	.82	.38	.29	− 4.11	.25
DM_10	.96	.19	1.60	− 2.55	.25
DM_11	.80	.40	1.11	− 1.10	.25
DM_12	.94	.25	2.07	− 1.80	.25
DM_13	.91	.28	1.25	− 2.06	.25
DM_14	.99	.12	1.80	− 3.08	.25
DM_15	.44	.50	1.21	1.09	.25
DM_16	.50	.50	− .09	− 7.85	.25
DM_17	.68	.47	.87	− .41	.25
DM_18	.73	.45	1.09	− .64	.25
DM_19	.13	.34	.16	66.76	.25
P_1	.94	.24	1.23	− 2.01	.50
P_2	.18	.38	− .29	− 38.23	.50
P_3	.91	.28	2.84	− 1.12	.50
P_4	.86	.35	1.23	− 1.00	.50
P_5	.83	.38	1.38	− .63	.50
P_6	.87	.33	1.37	− 1.07	.50
P_7	.86	.35	1.35	− .94	.50
P_8	.85	.36	1.37	− .75	.50
P_9	.90	.31	2.04	− 1.04	.50
P_10	.91	.29	3.10	− 1.05	.50
P_11	.92	.27	.92	− 2.09	.50
P_12	.92	.26	1.48	− 1.53	.50
P_13	.66	.47	.96	.94	.50
P_14	.53	.50	2.32	1.85	.50
P_15	.95	.22	1.92	− 1.68	.50
P_16	.95	.23	5.24	− 1.28	.50
P_17	.83	.38	2.15	− .46	.50
P_18	.82	.38	1.49	− .59	.50
P_19	.82	.38	.29	− 2.04	.50
P_20	.76	.43	1.37	− .13	.50
P_21	.72	.45	1.22	.27	.50
P_22	.92	.28	1.76	− 1.34	.50
P_23	.71	.45	1.48	.22	.50
P_24	.60	.49	1.26	1.28	.50
P_25	.89	.32	1.55	− 1.09	.50
P_26	.87	.33	2.04	− .93	.50

This table uses the following abbreviations for each item type: backwards reasoning (BR), *b*) different materials (DM), and *c*) pulling (*P*)

**Table 8:** Study 1: item response theory 2PL model—item-level information about knot reasoning task (shortened—54 items)

Item	Proportion correct	Standard deviation	Discrimination parameter (*a*)	Difficulty parameter (*b*)	Guessing parameter (*g*)
BR_1	.59	.49	1.20	.16	.25
BR_5	.42	.49	2.97	.71	.25
BR_7	.73	.44	1.17	− .63	.25
BR_8	.60	.49	1.84	.15	.25
BR_10	.63	.48	1.23	− .05	.25
BR_11	.80	.40	1.43	− .93	.25
BR_12	.63	.48	4.29	− .02	.25
BR_13	.71	.45	1.70	− .40	.25
BR_14	.66	.47	1.95	− .14	.25
BR_15	.72	.45	1.94	− .38	.25
BR_16	.73	.44	1.41	− .57	.25
BR_17	.55	.50	2.04	.31	.25
BR_18	.71	.46	1.26	− .44	.25
BR_19	.74	.44	1.68	− .52	.25
BR_21	.62	.49	1.51	.01	.25
BR_22	.57	.50	1.35	.32	.25
BR_25	.42	.49	1.11	1.45	.25
BR_26	.58	.49	1.75	.21	.25
BR_27	.67	.47	1.70	− .25	.25
BR_28	.47	.50	1.38	.88	.25
DM_2	.79	.41	1.53	− .87	.25
DM_4	.98	.13	3.09	− 2.42	.25
DM_6	.96	.19	1.14	− 3.14	.25
DM_7	.96	.19	2.88	− 1.92	.25
DM_8	.88	.32	1.31	− 1.67	.25
DM_10	.96	.19	1.61	− 2.55	.25
DM_11	.80	.40	1.10	− 1.11	.25
DM_12	.94	.25	2.12	− 1.79	.25
DM_13	.91	.28	1.22	− 2.09	.25
DM_14	.99	.12	1.82	− 3.06	.25
DM_15	.44	.50	1.23	1.09	.25
DM_18	.73	.45	1.10	− .64	.25
P_1	.94	.24	1.23	− 2.01	.50
P_3	.91	.28	2.85	− 1.13	.50
P_4	.86	.35	1.18	− 1.02	.50
P_5	.83	.38	1.37	− .64	.50
P_6	.87	.33	1.43	− 1.05	.50
P_7	.86	.35	1.28	− .97	.50
P_8	.85	.36	1.34	− .76	.50
P_9	.90	.31	1.93	− 1.07	.50
P_10	.91	.29	3.24	− 1.05	.50
P_12	.92	.26	1.50	− 1.53	.50
P_14	.53	.50	4.02	1.64	.50
P_15	.95	.22	1.81	− 1.75	.50
P_16	.95	.23	4.98	− 1.30	.50
P_17	.83	.38	2.03	− .47	.50
P_18	.82	.38	1.46	− .60	.50
P_20	.76	.43	1.43	− .13	.50
P_21	.72	.45	1.23	.27	.50
P_22	.92	.28	1.74	− 1.36	.50
P_23	.71	.45	1.55	.22	.50
P_24	.60	.49	1.23	1.30	.50
P_25	.89	.32	1.54	− 1.10	.50
P_26	.87	.33	1.97	− .94	.50

This table uses the following abbreviations for each item type: backwards reasoning (BR), **b**) different materials (DM), and **c**) pulling (*P*)

**Table 9:** Study 2: item response theory 1PL model—item-level information about knot reasoning task (shortened—54 items)

Item	Proportion correct	Standard deviation	Discrimination parameter (*a*)	Difficulty parameter (*b*)	Guessing parameter (*g*)
BR_1	.67	.47	1	− .27	.25
BR_5	.67	.47	1	− .23	.25
BR_7	.87	.34	1	− 1.93	.25
BR_8	.75	.44	1	− .87	.25
BR_10	.76	.43	1	− .97	.25
BR_11	.91	.28	1	− 2.43	.25
BR_12	.84	.37	1	− 1.60	.25
BR_13	.86	.35	1	− 1.78	.25
BR_14	.73	.45	1	− .75	.25
BR_15	.84	.36	1	− 1.69	.25
BR_16	.86	.34	1	− 1.87	.25
BR_17	.66	.48	1	− .31	.25
BR_18	.87	.34	1	− 1.88	.25
BR_19	.86	.34	1	− 1.82	.25
BR_21	.85	.36	1	− 1.75	.25
BR_22	.68	.47	1	− .33	.25
BR_25	.43	.50	1	1.42	.25
BR_26	.75	.44	1	− .84	.25
BR_27	.82	.39	1	− 1.36	.25
BR_28	.53	.50	1	.65	.25
DM_2	.88	.33	1	− 1.97	.25
DM_4	.99	.08	1	− 5.28	.25
DM_6	.97	.16	1	− 3.81	.25
DM_7	.98	.14	1	− 4.14	.25
DM_8	.98	.14	1	− 4.13	.25
DM_10	.99	.12	1	− 4.56	.25
DM_11	.86	.34	1	− 1.87	.25
DM_12	.97	.16	1	− 3.82	.25
DM_13	.95	.23	1	− 3.05	.25
DM_14	1.00	NA	NA	NA	NA
DM_15	.56	.50	1	.52	.25
DM_18	.91	.28	1	− 2.43	.25
P_1	.98	.14	1	− 3.70	.50
P_3	.95	.21	1	− 2.71	.50
P_4	.84	.36	1	− .92	.50
P_5	.86	.34	1	− 1.24	.50
P_6	.92	.27	1	− 1.94	.50
P_7	.86	.35	1	− 1.24	.50
P_8	.86	.35	1	− 1.18	.50
P_9	.95	.21	1	− 2.65	.50
P_10	.92	.27	1	− 1.97	.50
P_12	.95	.21	1	− 2.64	.50
P_14	.65	.48	1	1.32	.50
P_15	.98	.14	1	− 3.66	.50
P_16	.94	.24	1	− 2.37	.50
P_17	.74	.44	1	− .06	.50
P_18	.82	.38	1	− .69	.50
P_20	.93	.26	1	− 2.09	.50
P_21	.73	.44	1	.30	.50
P_22	.93	.25	1	− 2.24	.50
P_23	.82	.38	1	.− .65	.50
P_24	.64	.48	1	.92	.50
P_25	.89	.31	1	1.43	.50
P_26	.93	.26	1	− 2.12	.50

This table uses the following abbreviations for each item type: backwards reasoning (BR), *b*) different materials (DM), and *c*) pulling (*P*)

*NA* not applicable (not included in IRT models due to zero variance in performance on this item)

## Appendix C: Spatial activities questionnaire items

**Table:**

Neutral	Masculine-stereotyped	Feminine-stereotyped	New Fiber Arts Items
Bowling	Tackle Football	Figure Skating	Crochet (without seams)
Softball	Baseball	Baton Twirling (with tossing in air)	Embroidery, needlepoint (with pattern)
Tennis—serve and volley; place shots	Target-shooting (pistol or rifle)	Gymnastics	Knit (single-color pattern)
Racquetball	Building model trains or racing car sets	Ballet (with pirouettes, turns with spotting)	Sew (clothes and other items)
Ping-Pong (table tennis)	Making or fixing radios, stereos	Embroidery, needlepoint not following printed pattern	Macramé
Volleyball	Building go-carts or soapbox cars	Crochet (pieces needing seams)	Rug hooking
Dodgeball	Carpentry	Knitting multi-color patterns	Make friendship bracelets
Diving	Electrical repairs	Making patchwork quilts	Weave (e.g., with a loom)
Painting (in 3-D perspective)	Car repair	Sketching clothes designs	Braid hair
Drawing (in 3-D perspective)	Touch Football	Interior decorating (planning furniture, colors, decoration, etc. for room)	Make lace
			Spin yarn
			Dye fabric

## Appendix D: Supplemental analyses of spatial activities questionnaire—30 items

In order to facilitate comparisons to prior work, we reanalyzed our Spatial Activities Questionnaire (SAQ) and childhood SAQ using the 30 items that were the same as prior studies (Signorella et al., 1986). In the main text, we added 12 fiber-arts-related items to the original 30-item SAQ, totaling 42 items. Because our new items were all part of the feminine-stereotyped subscale, this reanalysis using the original 30 items changed the overall scale and the feminine-stereotyped subscale, but not the masculine-stereotyped subscale. Descriptive statistics for the original 30-item SAQ and childhood SAQ are reported in Table 10, and correlations among these scales and cognitive measures are reported in Table 11. Gender differences in these measures are depicted in Fig. 13.

Using the original 30-item scale, there was not a significant difference in overall current SAQ or childhood SAQ for men versus women when we used a Benjamini–Hochberg correction for multiple comparisons (current: *t*(105.81) = − 0.75, corrected *p* = 0.455, *d* = 0.13; childhood: *t*(110.04) = − 1.42, corrected *p* = 0.191, *d* = 0.25). When we looked at only the original 10 feminine-stereotyped items, we again found that women reported engaging in these activities significantly more often than men (current: *t*(132.39) = 4.01, corrected *p* < 0.001, *d* = − 0.66; childhood: *t*(131.57) = 6.68, corrected *p* < 0.001, *d* = − 1.05).

When we ran the SEMs with the 30-item SAQ, gender no longer predicted overall SAQ (Model D.2, Fig. 14). Otherwise, we found the same pattern for the other cognitive measures, where gender predicted performance on the mental rotation task, paper folding task, knot reasoning task, and Wordsum task (0.025 < *p* < 0.001), but not the bending task (*β* = 0.21, SE = 0.16,* p* = 0.191). In Model D.3 (Fig. 15), we found a similar pattern as when we ran the model with the expanded SAQ. However, unlike the model with the expanded SAQ, in the model using the 30-item SAQ, men did not significantly outperform women on the bending task (*β* = 0.36, SE = 0.20,* p* = 0.068), though the direction and size of the effect were similar. Like the model with the expanded SAQ, masculine-stereotyped current spatial activities were negatively associated with mental rotation task performance (*β* = − 0.38, SE = 0.16,* p* = 0.020) and the indirect effect was significant (*β* = − 0.19, 95% CI [− 0.38, − 0.06]). Further, feminine-stereotyped current spatial activities were again positively associated with paper folding task performance (*β* = 0.37, SE = 0.14,* p* = 0.007) and the indirect effect was significant (*β* = − 0.16, 95% CI [− 0.31, − 0.03]). Unlike the model with the expanded SAQ, we did not see that masculine-stereotyped spatial skills were significantly related to performance on the knot reasoning task (*β* = − 0.33, SE = 0.17, *p* = 0.059), though again, the direction and magnitude of the effect were similar to our main analyses.

In Model D.4 (Fig. 16), using the 30-item childhood SAQ, we found some similarities and some differences from the pattern of results in the main text. First, we found that there was no longer a significant direct effect of gender on Wordsum performance (*β* = 0.39, SE = 0.21, *p* = 0.056), though the effect was similar in magnitude and direction. Next, we found that feminine-stereotyped spatial activities in childhood predicted paper folding task performance, which is the same as our main text results with the expanded SAQ (*β* = 0.28, SE = 0.10,* p* = 0.006). In addition, this model showed that childhood feminine-stereotyped spatial activities significantly predicted bending task performance (*β* = 0.21, SE = 0.10,* p* = 0.025), a path that was not significant in our main analyses. Feminine-stereotyped spatial activities mediated the relationship between gender and paper folding task (*β* = − 0.24, 95% CI [− 0.43, − 0.06]) and bending task (*β* = − 0.18, 95% CI [− 0.36, − 0.02]) (Tables 10, 11 and Figs. 13, 14, 15, 16, 17, 18, 19, 20, 21, 22, 23, 24, 25).

**Table 10:** Descriptive statistics for activities scores

	All participants (*n* = 147)	Men (*n* = 56)	Women (*n* = 82)	Non-binary people (*n* = 9)
Variables	*M* (SD)	*M* (SD)	*M* (SD)	*M* (SD)
SAQ (30 items)	1.37 (.26)	1.40 (.28)	1.36 (.25)	1.29 (.26)
SAQ feminine (10 items)	1.26 (.29)	1.14 (.21)	1.31 (.28)	1.44 (.49)
C-SAQ (30 items)	3.50 (1.95)	3.80 (2.03)	3.31 (1.89)	3.39 (1.90)
C-SAQ feminine (10 items)	2.08 (2.25)	.79 (1.27)	2.81 (2.25)	3.33 (3.33)

*SAQ* Spatial Activities Questionnaire, *SAQ feminine* Spatial Activities Questionnaire Feminine-Stereotyped Items, *C-SAQ* Childhood Spatial Activities Questionnaire, *C-SAQ feminine* Childhood Spatial Activities Questionnaire Feminine-Stereotyped Items

**Table 11:** Correlations among the cognitive and activities scores—30 items

	1	2	3	4	5	6	7	8	9	10
1. MRT										
2. PFT	.53***									
3. BT	.31***	.47***								
4. KRT	.54***	.59***	.33***							
5. WS	.28**	.28**	.04	.29***						
6. SAQ (30 items)	− .02	− .01	− .05	− .13	.02					
7. SAQ masculine	− .01	− .05	.00	− .07	.13	.57***				
8. SAQ feminine (10 item)	.00	.14	.10	.05	.02	.51***	− .04			
9. C-SAQ (30 items)	.14	.14	.18*	.06	.08	.50***	.35***	.22**		
10. C-SAQ masculine	.15	.08	.11	.01	.12	.39***	.70***	− .13	.56***	
11. C-SAQ feminine (10 item)	− .03	.13	.10	.05	− .06	.29***	− .15	.59***	.49***	− .17*

****p* < .001, ***p* < .01, **p* < .05

*MRT* Mental rotation task, *PFT* paper folding task, *BT* bending task, *KRT* knot reasoning task, *WS* Wordsum Plus, *SAQ* Spatial Activities Questionnaire, *SAQ masculine* Spatial Activities Questionnaire Masculine-Stereotyped Items, *SAQ feminine* Spatial Activities Questionnaire Feminine-Stereotyped Items, *C-SAQ* Childhood Spatial Activities Questionnaire, *C-SAQ masculine* Childhood Spatial Activities Questionnaire Masculine-Stereotyped Items, *C-SAQ feminine* Childhood Spatial Activities Questionnaire Feminine-Stereotyped Items

## Appendix E: Major coding scheme (adapted from Tian et al., 2023)

See Table 12.

**Table 12:** Categorization of STEM or non-STEM field of study

Field of study (in SECCYD)	Total number of major programs*	Number of STEM major programs*	Proportion of STEM programs	Category
Philosophy and religious studies	14	0	0.00	Non-STEM
Area, ethnic, cultural, and gender studies	46	0	0.00	
Public administration and social service professions	12	0	0.00	
Theology and religious vocations	17	0	0.00	
Construction	26	0	0.00	
Visual and performing arts	64	0	0.00	
English language and literature	13	0	0.00	
Family and consumer sciences/human sciences	34	0	0.00	
Foreign languages, literature, and linguistics	82	0	0.00	
History	9	0	0.00	
Legal professions and studies	21	0	0.00	
Liberal arts and sciences, general studies and humanities	4	0	0.00	
Library science	6	0	0.00	
Mechanic and repair technologies/technicians	38	0	0.00	
Parks, recreation, leisure, fitness studies	12	0	0.00	
Personal and culinary services	30	0	0.00	
Education	100	3	0.03	
Business, management, marketing, and related support services	94	4	0.04	
Communication, journalism, and related programs	23	1	0.04	
Security and protective services	34	2	0.06	
Communications technologies/technicians and support services	15	1	0.07	
Health professions and related clinical sciences	237	20	0.08	
Architecture and related services	11	1	0.09	
Social sciences	32	3	0.09	
Psychology	27	10	0.37	STEM
Agriculture, agriculture operations, and related sciences	62	23	0.37	
Multi-/interdisciplinary studies	32	13	0.41	
Natural resources and conservation	22	9	0.41	
Computer and information sciences and support services	30	26	0.87	
Physical sciences	43	43	1.00	
Biological and biomedical sciences	89	89	1.00	
Science technologies/technicians	9	9	1.00	
Engineering	53	53	1.00	
Mathematics and statistics	17	17	1.00	
Military technology	27	27	1.00	

*The total number of major programs in each field of study was calculated according to the NCES CIP list. The number of STEM major programs in each field of study was calculated according to the DHS STEM Program List

Based on our coding criterion, Health Professions and Related Clinical Sciences should be categorized as a non-STEM field of study because 9% of the major programs within this field were considered as STEM programs by DHS. However, several prior studies on college major choices grouped Health Professions with typical STEM fields of studies (Perez-Felkner et al., 2017). We then referred to the American Freshmen survey (Eagan et al., 2017), which reported data that were statistically weighted to reflect characteristics of approximately 1.5 million college freshmen entering 4-year colleges across the USA in the fall of 2016. The survey showed that nearly half of college freshmen who intended to purse a major within the field of health professions indicated nursing or therapy as their intended major, neither of which was considered as a STEM major by DHS. Therefore, we categorized Health Professions and Related Clinical Sciences as a non-STEM field of study.

Among the 35 fields of study that were included in the SECCYD dataset, 24 fields were considered as non-STEM fields, and 11 were considered as STEM fields. Within STEM fields, we further categorized each field of study into (1) math-intensive fields or (2) non-math-intensive fields (see Table 13 below), based on math-intensiveness coding of college majors in prior research (Goldman & Hewitt, 1976).

**Table 13:** Math-intensiveness of STEM fields of study

Field of study (in SECCYD)	Math-intensiveness
Psychology	Non-math-intensive STEM
Agriculture, agriculture operations, and related sciences	
Multi-/interdisciplinary studies	
Natural resources and conservation	
Biological and biomedical sciences	
Science technologies/technicians	
Military technology	
Computer and information sciences and support services	Math-intensive STEM
Physical sciences	
Engineering	
Mathematics and statistics	

For each participant, we coded each of their fields of study based on the above-described scheme: First determine whether the field of study is a STEM or non-STEM field of study; then, if the field of study is a STEM one, further determine whether the field of study is math-intensive or non-math-intensive. For participants choosing multiple fields of study, each field of study was coded separately, and these participants were categorized based on their most math-intensive major (math-intensiveness: math-intensive STEM > non-math-intensive STEM > non-STEM).

For participants who wrote in their field of study, we first searched for the written major program name in the 2010 NCES CIP list and the corresponding field of study. We referred to the program description if the name was ambiguous (e.g., “piano performance” is coded as program “Keyboard Instruments” and field “Visual and Performing Arts”). Then, we coded the field of study using the same scheme as described above. Three new fields, which are not in the SECCYD dataset, were added to the current coding scheme: “Engineering/Engineering-Related Technologies/Technicians” (STEM and math-intensive), “Precision Production” (non-STEM), “Transportation and Materials Moving” (non-STEM).

Additionally, Table 14 that may be helpful for the math-intensive versus non-math-intensive choice is given.

**Table 14:** Composition of science–nonscience categories

1 Fine arts	2 Humanities	3 Social Science	4 Biological Science	5 Physical Science
Art, Studio	English	Agricultural Economics and Business Management	Animal Science	Applied Mechanics and Engineering Science
Dance	French	Anthropology	Bacteriology	Applied Physics and Informational Sciences
Design	History	Child Development	Biochemistry	Chemistry
Drama	English and American Literature	Comparative Cultures	Biological Science	Engineering, Lower Division
Music	Spanish	Economics	Biology	Electrical Engineering
Paint/Sculpture and Graphic Art		Environmental Planning and Management	Dietetics	Information and Computer Sciences
Theater		Home Economics	Food Science	Mathematics
		Individual group	Kinesiology	Mathematics and Computer Science
		International Relations	Physiology	Physics
		Political Science	Plant Science	Prepharmaceutics
		Precriminology	Prenursing	
		Prepsychology	Wildlife Fisheries Biology	
		Psychology	Zoology	
		Social Science		
		Sociology		

Many major fields which clearly belong in these categories were omitted because they contained too few students (*n* < 60)

Notes for this coding:

1. if participant has “undecided” or “DUS,” leave it blank.

2. Nursing/health professions = 0.

3. for math-intensive v. non-math-intensive coding, biochemistry = 0, bioengineering = 1.

4. pharm.d = 1 for both layers of coding.

## Footnotes

1 Prolific uses the following categories, recommended by the UK Office of National Statistics, to balance based on race: White, Mixed, Asian, Black, and Other. We asked participants to report their race based on the US Census categories, which we report here.

2 Prolific asks all users to answer a set of standardized demographic questions, including their sex (male or female) and their gender (woman, man, non-binary, other). Prolific can then use responses to recruit an equal number of participants who respond “male” and “female.” However, this does not exclude participants who answer that they are non-binary on the separate question about gender.

3 Unfortunately, we do not have item-level timing data.

4 We also include the item characteristic curves for the 73-item and 54-item 2PL models in Appendix B, Fig. 10, as well as the standard error plots for the 73-item and 54-item 2PL models in Appendix B, Fig. 9.

5 One participant was excluded from the analytic sample due to excessive sleepiness during the study session.

6 The same pattern held true for men and women who were current undergraduates (Men: *M* = 20.5, SD  2.47; Women: *M* = 19.6, SD 1.37; *t*(103.60) = − .80, *p* = .428, *d* = .15).

7 In order to facilitate comparisons to prior work, we also ran all of the models that include the overall spatial activities score with the original 30 items and report these in Appendix D. Reliability for the original SAQ was similar (overall *α* = .67, feminine-stereotyped items *α* = .45). Results were similar to those reported in the main text, though notably gender no longer predicted overall SAQ score when using the original 30 items.

8 Two prior studies (Peterson et al., 2020; Signorella et al., 1986) took a different approach and summed the responses for all activities included in the original SAQ (minimum score of 30, maximum score of 180) to make a total activities score, as well as creating masculine- and feminine-stereotyped activities scores (minimum score of 10, maximum score of 60). Another study (Nazareth et al., 2013) gave 1 point for each masculine-stereotyped activity that participants endorsed (range from 0 to 40) and 1 point for each “other” (feminine-stereotyped + neutral) activity that participants endorsed (range from 0 to 41).

9 If any participants responded something other than “Never” for childhood frequency, but then responded “Never” for childhood intensity, we treated this as a “Never” for frequency. This was the case for two items across two participants.

10 There was one outlier on the bending task, one on the Wordsum task, one on all spatial activities except for childhood masculine-stereotyped spatial activities, two on current masculine-stereotyped spatial activities, two on current feminine-stereotyped spatial activities, and one on both current and childhood feminine-stereotyped spatial activities.

11 Given this surprising finding, we ran supplemental analyses to check that the gender differences in task performance could be explained by men being older than women in our sample. We ran five regressions with age and gender (only men and women) predicting performance on each cognitive measure and found that age was not a significant predictor. Gender remained a significant predictor for the mental rotation, paper folding, Wordsum Plus, and knot reasoning tasks.

12 We did not know how many participants would identify as non-binary in our sample, and we anticipated that this number would be substantially smaller than the number of participants identifying as men or women. We planned to run Welch’s between-groups t-tests comparing non-binary participants and women, and non-binary participants and men if we had 10 or more participants identifying as non-binary; however, we did not meet this threshold.

13 We also ran an ANOVA to check that age did not significantly differ by major, and found that it did not (*F*(2, 140) = 1.37, *p* = .258, *η*^2^ = .02).

## References

[CR1] Atit, K., Shipley, T. F., & Tikoff, B. (2013). Twisting space: Are rigid and non-rigid mental transformations separate spatial skills? *Cognitive Processing,**14*(2), 163–173. 10.1007/s10339-013-0550-8 23423639 10.1007/s10339-013-0550-8

[CR2] Bennett, G. K., Seashore, H. G., & Wesman, A. G. (1947). *Differential aptitude tests*. Psychological Corporation.

[CR3] Bennett-Pierre, G., & Gunderson, E. A. (2023). Fiber arts require spatial skills: How a stereotypically feminine practice can help us understand spatial skills and improve spatial learning. *Sex Roles,**88*(1–2), 1–16. 10.1007/s11199-022-01340-y

[CR4] Bentler, P., & Chou, C.-P. (1987). Practical issues in structural modeling. *Sociological Methods & Research,**16*(1), 78–117. 10.1177/0049124187016001004

[CR5] Brandt, M. G., & Davies, E. T. (2006). Visual-spatial ability, learning modality and surgical knot tying. *Canadian Journal of Surgery,**49*(6), 412–416. PMC3207546 17234070

[CR7] Casasola, M., Wei, W. S., Suh, D. D., Donskoy, P., & Ransom, A. (2020). Children’s exposure to spatial language promotes their spatial thinking. *Journal of Experimental Psychology: General,**149*(6), 1116–1136. 10.1037/xge0000699 32212765 10.1037/xge0000699

[CR8] Casey, B. M., Andrews, N., Schindler, H., Kersh, J. E., Samper, A., & Copley, J. (2008). The development of spatial skills through interventions involving block building activities. *Cognition and Instruction,**26*(3), 269–309. 10.1080/07370000802177177

[CR9] Chatterjee, A. (2008). The neural organization of spatial thought and language. In *Seminars in speech and language* (Vol. 29, No. 03, pp. 226–238). Thieme Medical Publishers. 10.1055/s-0028-1082886 10.1055/s-0028-1082886 18720319

[CR10] Cherney, I. D., & Voyer, D. (2010). Development of a spatial activity questionnaire I: Items identification. *Sex Roles,**62*(1–2), 89–99. 10.1007/s11199-009-9710-9

[CR11] Cor, M. K., Haertel, E., Krosnick, J. A., & Malhotra, N. (2012). Improving ability measurement in surveys by following the principles of IRT: The Wordsum vocabulary test in the General Social Survey. *Social Science Research,**41*(5), 1003–1016. 10.1016/j.ssresearch.2012.05.007 23017913 10.1016/j.ssresearch.2012.05.007

[CR12] Coyle, E. F., & Liben, L. S. (2020). Gendered packaging of a STEM toy influences children’s play, mechanical learning, and mothers’ play guidance. *Child Development,**91*(1), 43–62. 10.1111/cdev.13139 30187910 10.1111/cdev.13139

[CR13] DeMars, C. (2010). Chapter 2: Requirements. In *Item response theory* (pp. 31–37). Oxford University Press.

[CR14] Doyle, R. A., Voyer, D., & Cherney, I. D. (2012). The relation between childhood spatial activities and spatial abilities in adulthood. *Journal of Applied Developmental Psychology,**33*(2), 112–120. 10.1016/j.appdev.2012.01.002

[CR63] Eagan, M. K., Stolzenberg, E. B., Zimmerman, H. B., Aragon, M. C., Sayson, H. W., & Rios-Aguilar, C. (2017). *The American freshman: National norms fall 2016*. Los Angeles: Higher Education Research Institute, UCLA.

[CR15] Ekstrom, R. B. (1976). *Kit of factor-referenced cognitive tests*. Educational Testing Service.

[CR16] Fehringer, B. (2020). Spatial thinking from a different view: Disentangling top-down and bottom-up processes using eye tracking. *Open Psychology,**2*(1), 138–212. 10.1515/psych-2020-0105

[CR17] Fein, E., Gilmour, J., Machin, T., & Hendry, L. (2022). Exploring your data: Outliers. In *Statistics for research students: An open access resource with self-tests and illustrative examples*. University of South Queensland.

[CR64] Goldman, R. D., Hewitt, B. N. (1976). The Scholastic Aptitude Test explains why college men major in science more often than college women. *Journal of Counseling Psychology*, *23*(1), 50–54. 10.1037/0022-0167.23.1.50.

[CR18] Gresalfi, M., & Chapman, K. (2017). Recrafting manipulatives: Toward a critical analysis of gender and mathematical practice. *Mathematics Education and Life at times of Crisis,**2*, 491.

[CR19] Grogono, A. (1996). *Animated Knots*. Animated Knots. https://www.animatedknots.com/

[CR20] Harris, J., Hirsh-Pasek, K., & Newcombe, N. S. (2013). Understanding spatial transformations: Similarities and differences between mental rotation and mental folding. *Cognitive Processing,**14*(2), 105–115. 10.1007/s10339-013-0544-6 23397105 10.1007/s10339-013-0544-6

[CR21] Hawes, Z. C. K., Gilligan-Lee, K. A., & Mix, K. S. (2022). Effects of spatial training on mathematics performance: A meta-analysis. *Developmental Psychology,**58*(1), 112–137. 10.1037/dev0001281 35073120 10.1037/dev0001281

[CR22] Hegarty, M., & Waller, D. A. (2005). Individual differences in spatial abilities. In *The Cambridge handbook of visuospatial thinking* (pp. 121–169). Cambridge University Press.

[CR23] Hyde, J. S., & Linn, M. C. (1988). Gender differences in verbal ability: A meta-analysis. *Psychological Bulletin,**104*(1), 53–69. 10.1037/0033-2909.104.1.53

[CR24] Jirout, J. J., & Newcombe, N. S. (2015). Building blocks for developing spatial skills: Evidence from a large, representative U.S. sample. *Psychological Science,**26*(3), 302–310. 10.1177/0956797614563338 25626442 10.1177/0956797614563338

[CR25] Johnson, T., Burgoyne, A. P., Mix, K. S., Young, C. J., & Levine, S. C. (2022). Spatial and mathematics skills: Similarities and differences related to age, SES, and gender. *Cognition,**218*, 104918. 10.1016/j.cognition.2021.104918 34627067 10.1016/j.cognition.2021.104918

[CR26] Keehner, M. M., Tendick, F., Meng, M. V., Anwar, H. P., Hegarty, M., Stoller, M. L., & Duh, Q.-Y. (2004). Spatial ability, experience, and skill in laparoscopic surgery. *The American Journal of Surgery,**188*(1), 71–75. 10.1016/j.amjsurg.2003.12.059 15219488 10.1016/j.amjsurg.2003.12.059

[CR27] Lauer, J. E., Yhang, E., & Lourenco, S. F. (2019). The development of gender differences in spatial reasoning: A meta-analytic review. *Psychological Bulletin,**145*(6), 537–565. 10.1037/bul0000191 30973235 10.1037/bul0000191

[CR28] Levine, S. C., Huttenlocher, J., Taylor, A., & Langrock, A. (1999). Early sex differences in spatial skill. *Developmental Psychology,**35*(4), 940–949. 10.1037/0012-1649.35.4.940 10442863 10.1037//0012-1649.35.4.940

[CR29] Lietz, P. (2006). A meta-analysis of gender differences in reading achievement at the secondary school level. *Studies in Educational Evaluation,**32*(4), 317–344. 10.1016/j.stueduc.2006.10.002

[CR30] MacCallum, R. C., Browne, M. W., & Cai, L. (2006). Testing differences between nested covariance structure models: Power analysis and null hypotheses. *Psychological Methods,**11*(1), 19–35. 10.1037/1082-989X.11.1.19 16594765 10.1037/1082-989X.11.1.19

[CR31] Maheshwary, K., Eisenbart, B., Zorn, S., Nelius, T., Gericke, K., Matthiesen, S., & Blessing, L. (2022). Assessing rigid and non-rigid spatial thinking. *Proceedings of the Design Society,**2*, 2333–2342. 10.1017/pds.2022.236

[CR32] Malhotra, N., Krosnick, J., & Haertel, E. (2007). The psychometric properties of the GSS wordsum vocabulary test. *GSS Methodological Report,**11*, 1–63.

[CR33] McNeal, P., & Petcovic, H. (2020). Spatial thinking and fluid Earth science education research. *Journal of Geoscience Education,**68*(4), 289–301. 10.1080/10899995.2020.1768007

[CR34] McNeal, P., Petcovic, H., Ladue, N., & Ellis, T. (2019). Identifying significant cognitive factors for practicing and learning meteorology. *Journal of Operational Meteorology,**7*(1), 1–26. 10.15191/nwajom.2019.0701

[CR35] Mix, K. S., & Cheng, Y.-L. (2012). The relation between space and math. In *Advances in child development and behavior* (Vol. 42, pp. 197–243). Elsevier. 10.1016/B978-0-12-394388-0.00006-X 10.1016/b978-0-12-394388-0.00006-x 22675907

[CR36] Mix, K. S., Hambrick, D. Z., Satyam, V. R., Burgoyne, A. P., & Levine, S. C. (2018). The latent structure of spatial skill: A test of the 2 × 2 typology. *Cognition,**180*, 268–278. 10.1016/j.cognition.2018.07.012 30098471 10.1016/j.cognition.2018.07.012

[CR37] Mix, K. S., Levine, S. C., Cheng, Y.-L., Stockton, J. D., & Bower, C. (2021). Effects of spatial training on mathematics in first and sixth grade children. *Journal of Educational Psychology,**113*(2), 304–314. 10.1037/edu0000494

[CR38] Munns, M., Tranquada-Torres, B., Chrasil, E., & Hegarty, M. (2022). Large-scale vs small-scale spatial abilities: Development of a broad spatial activities questionnaire. *Proceedings of the Annual Meeting of the Cognitive Science Society,**44*, 1079–1086.

[CR39] Nazareth, A., Herrera, A., & Pruden, S. M. (2013). Explaining sex differences in mental rotation: Role of spatial activity experience. *Cognitive Processing,**14*(2), 201–204. 10.1007/s10339-013-0542-8 23381194 10.1007/s10339-013-0542-8

[CR40] Newcombe, N. (2020). The puzzle of spatial sex differences: Current status and prerequisites to solutions. *Child Development Perspectives,**14*(4), 251–257. 10.1111/cdep.12389

[CR41] Newcombe, N., Bandura, M. M., & Taylor, D. G. (1983). Sex differences in spatial ability and spatial activities. *Sex Roles,**9*(3), 377–386. 10.1007/BF00289672

[CR42] Newcombe, N., & Shipley, T. (2015). Thinking about spatial thinking: New typology, new assessments. In J. S. Gero (Ed.), *Studying visual and spatial reasoning for design creativity* (pp. 179–192). Springer. 10.1007/978-94-017-9297-4_10

[CR43] Peppler, K., Keune, A., & Thompson, N. (2020). Reclaiming traditionally feminine practices and materials for STEM learning through the modern maker movement. In N. Holbert, M. Berland, & Y. B. Kafai (Eds.), *Designing constructionist futures* (pp. 127–140). The MIT Press. 10.7551/mitpress/12091.003.0017

[CR44] Peragine, D. E., Gervais, N. J., Simeon-Spezzaferro, C., & Einstein, G. (2022). A new angle on mental rotation ability in transgender men: Modulation by ovarian milieu. *Psychoneuroendocrinology,**141*, 105751. 10.1016/j.psyneuen.2022.105751 35398751 10.1016/j.psyneuen.2022.105751

[CR65] Perez-Felkner, L., Nix, S., & Thomas, K. (2017). Gendered pathways: How mathematics ability beliefs shape secondary and postsecondary course and degree field choices. *Frontiers in Psychology*, *8*, 218959. 10.3389/fpsyg.2017.00386 PMC5382838 28428762

[CR45] Peters, M., Laeng, B., Latham, K., Jackson, M., Zaiyouna, R., & Richardson, C. (1995). A redrawn Vandenberg and Kuse mental rotations test-different versions and factors that affect performance. *Brain and Cognition,**28*(1), 39–58. 7546667 10.1006/brcg.1995.1032

[CR46] Peterson, E. G., Weinberger, A. B., Uttal, D. H., Kolvoord, B., & Green, A. E. (2020). Spatial activity participation in childhood and adolescence: Consistency and relations to spatial thinking in adolescence. *Cognitive Research: Principles and Implications,**5*(1), 43. 10.1186/s41235-020-00239-0 32936362 10.1186/s41235-020-00239-0 PMC7494723

[CR47] Pruden, S. M., & Levine, S. C. (2017). Parents’ spatial language mediates a sex difference in preschoolers’ spatial-language use. *Psychological Science,**28*(11), 1583–1596. 10.1177/0956797617711968 28880726 10.1177/0956797617711968 PMC5673527

[CR48] Pruden, S. M., Levine, S. C., & Huttenlocher, J. (2011). Children’s spatial thinking: Does talk about the spatial world matter? Children’s spatial thinking. *Developmental Science,**14*(6), 1417–1430. 10.1111/j.1467-7687.2011.01088.x 22010900 10.1111/j.1467-7687.2011.01088.x PMC3372906

[CR49] R Core Team. (2021). *R: A language and environment for statistical computing.* [Computer software]. R Foundation for Statistical Computing.

[CR50] Resnick, I., & Shipley, T. F. (2013). Breaking new ground in the mind: An initial study of mental brittle transformation and mental rigid rotation in science experts. *Cognitive Processing,**14*(2), 143–152. 10.1007/s10339-013-0548-2 23440527 10.1007/s10339-013-0548-2

[CR51] Rosseel, Y. (2012). lavaan: An R package for structural equation modeling. *Journal of Statistical Software,**48*(2), 1–36.

[CR52] Santos, P. E., Cabalar, P., & Casati, R. (2019). The knowledge of knots: An interdisciplinary literature review. *Spatial Cognition & Computation,**19*(4), 334–358. 10.1080/13875868.2019.1667998

[CR53] Signorella, M. L., Krupa, M. H., Jamison, W., & Lyons, N. (1986). A short version of a spatial activity questionnaire. *Sex Roles,**14*(9–10), 475–479. 10.1007/BF00287448

[CR54] Thurstone, L. L., & Thurstone, T. G. (1947). *SRA primary mental abilities*. Science Research Associates.

[CR55] Tian, J., Ren, K., Newcombe, N. S., Weinraub, M., Vandell, D. L., & Gunderson, E. A. (2023). Tracing the origins of the STEM gender gap: The contribution of childhood spatial skills. *Developmental Science,**26*(2), e13302. 10.1111/desc.13302 35815802 10.1111/desc.13302 PMC10811757

[CR56] Tsigeman, E. S., Likhanov, M. V., Budakova, A. V., Akmalov, A., Sabitov, I., Alenina, E., Bartseva, K., & Kovas, Y. (2023). Persistent gender differences in spatial ability, even in STEM experts. *Heliyon,**9*(4), e15247. 10.1016/j.heliyon.2023.e15247 37101649 10.1016/j.heliyon.2023.e15247 PMC10123158

[CR57] Vandenberg, S. G., & Kuse, A. R. (1978). Mental rotations, a group test of three-dimensional spatial visualization. *Perceptual and Motor Skills,**47*(2), 599–604. 10.2466/pms.1978.47.2.599 724398 10.2466/pms.1978.47.2.599

[CR58] Verdine, B. N., Golinkoff, R. M., Hirsh-Pasek, K., Newcombe, N. S., & Bailey, D. (2017). Links between spatial and mathematical skills across the preschool years. *Monographs of the Society for Research in Child Development,**82*(1), 7–149. 10.1111/mono.12280 28181248 10.1111/mono.12280

[CR59] Verdine, B. N., Zimmermann, L., Foster, L., Marzouk, M. A., Golinkoff, R. M., Hirsh-Pasek, K., & Newcombe, N. (2019). Effects of geometric toy design on parent–child interactions and spatial language. *Early Childhood Research Quarterly,**46*, 126–141. 10.1016/j.ecresq.2018.03.015 30555211 10.1016/j.ecresq.2018.03.015 PMC6289199

[CR60] Voyer, D., Nolan, C., & Voyer, S. (2000). The relation between experience and spatial performance in men and women. *Sex Roles,**43*(11), 891–915. 10.1007/s11199-009-9710-9

[CR61] Voyer, D., Voyer, S., & Bryden, M. P. (1995). Magnitude of sex differences in spatial abilities: A meta-analysis and consideration of critical variables. *Psychological Bulletin,**117*(2), 250–270. 10.1037/0033-2909.117.2.250 7724690 10.1037/0033-2909.117.2.250

[CR62] Wechsler, D. (2008). Wechsler adult intelligence scale—Fourth edition (WAIS-IV). *APA PsycTests*. 10.1037/t15169-000

